# Genome‐wide association study of resistance to PstS2 and Warrior races of *Puccinia striiformis f. sp. tritici* (stripe rust) in bread wheat landraces

**DOI:** 10.1002/tpg2.20066

**Published:** 2020-12-07

**Authors:** Muhammad Massub Tehseen, Fatma Aykut Tonk, Muzaffer Tosun, Ahmed Amri, Carolina P. Sansaloni, Ezgi Kurtulus, Mariana Yazbek, Khaled Al‐Sham'aa, Izzet Ozseven, Luqman Bin Safdar, Ali Shehadeh, Kumarse Nazari

**Affiliations:** ^1^ Department of Field Crops Ege University Izmir Turkey; ^2^ ICARDA‐PreBreeding & Genebank Operations Biodiversity and Crop Improvement Program Rabat Morocco; ^3^ Genetic Resources Program CIMMYT Mexico; ^4^ Turkey‐ICARDA Regional Cereal Rust Research Center (RCRRC) P.O. Box 35661 Menemen Izmir Turkey; ^5^ ICARDA‐Genetic Resources PreBreeding & Genebank Operations, Biodiversity and Crop Improvement Program Terbol Lebanon; ^6^ Geoinformatics & Research Data Management ICARDA Beirut Lebanon; ^7^ Agean Agricultural Research Institute Regional Cereal Rust Research Center (RCRRC) P.O. Box 35661 Menemen Izmir Turkey; ^8^ Department of Plant Sciences Quaid‐i‐Azam University Islamabad Pakistan

## Abstract

Stripe or yellow rust, caused by *Puccinia striiformis* Westend. f. sp. *tritici* is a major threat to bread wheat production worldwide. The breakdown in resistance of certain major genes and newly emerging aggressive races of stripe rusts pose serious concerns in all main wheat growing areas of the world. To identify new sources of resistance and associated QTL for effective utilization in future breeding programs an association mapping (AM) panel comprising of 600 bread wheat landraces collected from eight different countries conserved at ICARDA gene bank were evaluated for seedling and adult plant resistance against the *PstS2* and *Warrior* races of stripe rust at the Regional Cereal Rust Research Center (RCRRC), Izmir, Turkey during 2016, 2018 and 2019. A set of 25,169 informative SNP markers covering the whole genome were used to examine the population structure, linkage disequilibrium and marker‐trait associations in the AM panel. The genome‐wide association study (GWAS) was carried out using a Mixed Linear Model (MLM). We identified 47 SNP markers across 19 chromosomes with significant SNP‐trait associations for both seedling stage and adult plant resistance. The threshold of significance for all SNP‐trait associations was determined by the false discovery rate (q) ≤ 0.05. Three genomic regions (*QYr.1D_APR, QYr.3A_seedling* and *QYr.7D_seedling*) identified in this study do not correspond to previously reported *Yr* genes or QTL, suggesting new genomic regions for stripe rust resistance.

## Core Ideas

- The bread wheat landraces preserved in ICARDA gene bank are key genetic resource for resistance to biotic stresses
- Stripe rust is one of the most important disease of wheat
- Novel genomic regions identified harboring seedling and adult plant resistance against *PstS2* and *Warrior* races of stripe rust

## 1 INTRODUCTION

Wheat stripe rust, caused by *Puccinia striiformis* Westend. f. sp. *tritici* (*Pst*), is one of the most devastating diseases of bread wheat (*Triticum aestivum* L.) worldwide (Hovmøller, Sørensen, Walter, & Justesen, 2011). Historically, stripe rust is considered a disease of cool and wet climates, but with the emergence of new aggressive and high‐temperature tolerant pathotypes the disease is becoming a problem in areas once considered unfavorable for stripe rust infestation (Hovmøller et al., 2011; Muleta, Bulli, Rynearson, Chen, & Pumphrey, 2017; Sørensen, Hovmøller, Leconte, Dedryver, & de Vallavieille‐Pope, 2014). These new pathotypes of *Pst* are currently widespread from Asia to Europe, Africa and Australia threatening wheat yields at a global level (Ali et al., 2014). Stripe rust epidemics have been reported to cause yield losses up to 100% under severe infections (Manickavelu et al., 2016; Mumtaz et al., 2009).

The continuous emergence of new pathotypes of *Pst* demands the development of new varieties and strategies to resist the epidemic in time. The most economical and effective way of managing stripe rust outbreaks is the use of genetic resistance that combines both minor and major genes (Chen, 2013). Two types of genetic resistance based on major and minor genes are deployed in breeding programs all over the world (Chen, 2005). Resistance provided by major genes is often referred to as race‐specific, seedling and/or all‐stage resistance, which is based on the gene‐for‐gene hypothesis and is effective at all stages of plant life (Burdon, Barrett, Rebetzke, & Thrall, 2014). Commercial wheat varieties with resistance conferred by such major R genes are often short‐lived, as new virulent pathotypes continuously emerge in the pathogen populations which eventually overcome this resistance. Many devastating epidemics around the world are the result of such breakdowns of R gene‐mediated resistance in commercial varieties (Ellis, Lagudah, Spielmeyer, & Dodds, 2014; Hulbert & Pumphrey, 2014; Steele, Humphreys, Wellings, & Dickinson, 2001). On the other hand, minor gene resistance is usually not expressed until the adult plant stage, and referred to as horizontal/partial resistance or Adult Plant Resistance (APR). In many cases, pyramiding race‐specific major genes and/or slow‐rusting APR genes is considered in enhancing the durability of resistance to majority of the races of the pathogen, and hence minimizing yield losses compared to the complete yield loss in the case of breakdown of major gene resistance. Due to the rapid evolution of *Pst* pathogen, many pathotypes virulent to major resistance genes have emerged. Therefore, the best strategy is to pyramid effective multiple race‐specific major genes in combination with multiple non‐race specific minor genes in a variety to secure more durable rust resistance (Singh, Huerta‐Espino, & Rajaram, 2000, 2005).

The races belonging to *PstS2* and *Warrior* (*PstS7*) lineages are the most widely spread pathotypes of *Pst*, covering the geographical regions from Asia all the way to Northern Europe (Hovmøller et al., 2016; Tadesse et al., 2014). The *PstS7* lineage was first discovered in the UK in 2011, and currently is the most prevalent race of *Pst* in Europe (Ali et al., 2017). It was detected in Turkey in 2014 (Mert et al., 2016). Since then it has spread and widely established itself in North Africa (Hovmøller et al., 2016), and in the Middle East (K. Nazari, unpublished data). The ‘*Warrior’* type races has caused significant epidemics on many wheat varieties in Europe (Rahmatov, 2016). The breakdown of resistance gene *Yr27* in many countries in Central West Asia and North Africa (CWANA) caused significant yield losses. During the 2010 rust epidemics in Syria, which was due to the breakdown of resistance based on *Yr27* present in a widely grown bread wheat variety, ‘Cham 8’ (derived from the CIMMYT cross ‘Super Kauz’), losses of up to 80% were reported (Solh, Nazari, Tadesse, & Wellings, 2012). The development and deployment of genetic resistance to these two prevalent races of *Pst* is very crucial for the CWANA region. Deployment of combinations of major and minor genes is the long‐term goal of the breeding programs around the globe. This strategy thus provides resistance to the broader spectrum of races, thus providing wider durability against multiple virulent pathotypes (Chen, 2013; Singh et al., 2000). Recent advances in genomics and statistical approaches that provide effective molecular marker tagging systems have led to facilitating this strategy of pyramiding major and minor genes with higher efficiency (Bentley et al., 2014; Muleta et al., 2017).

Genome‐Wide Association Studies (GWAS) take advantage of historical recombination within the target crop resulting in population diversification, by determining Linkage Disequilibrium (LD) information within the gene pool of a species. This allows the identification of phenotype‐genotype correlations, and has been widely and successfully used to detect quantitative trait loci (QTL) in many plant species (Bulli, Zhang, Chao, Chen, & Pumphrey, 2016; Kertho, Mamidi, Bonman, McClean, & Acevedo, 2015; Maccaferri et al., 2015; Manickavelu et al., 2016; Muleta et al., 2017). The primary gene pool of wheat preserved in gene banks around the world, including landraces, traditional varieties and breeding lines, offers a diverse range of potential sources of resistance to biotic and abiotic stresses including disease resistance (Endresen et al., 2011). The co‐evolution of landraces along with biotic and abiotic stresses has made them an important choice in the selection of breeding/pre‐breeding materials for crop improvement (Zeven, 1998). Similarly, wheat landraces have co‐evolved with rust pathogens, which might have resulted in the accumulation of diverse resistance loci (Maccaferri et al., 2015). These landraces may possess useful untapped genetic resistance loci, since they have not been widely utilized in breeding programs. The use of germplasm preserved at gene banks for disease resistance in wheat has been previously reported in numerous studies (Bulli et al., 2016; Gurung et al., 2014; Kertho et al., 2015; Naruoka, Garland‐Campbell, & Carter, 2015; Maccaferri et al., 2015; Manickavelu et al., 2016; Muleta et al., 2017).

In this study, we examined the bread wheat landraces preserved at the gene bank of The International Center for Agriculture Research in the Dry Areas (ICARDA) as sources of resistance against important *Pst* races in CWANA. The present study has the following three objectives: (1) to evaluate the seedling and adult‐plant resistance to *Pst* pathotypes from *PstS2* and *PstS7* (*Warrior*) lineages in bread wheat landraces, (2) to perform GWAS analysis to identify single nucleotide polymorphism (SNP) loci associated with resistance to these two *Pst* pathotypes, and (3) to compare and evaluate the *Pst* resistance loci identified in this study with previously identified *Yr* genes and QTL.

## 2 MATERIALS AND METHODS

### 2.1 Plant material

A panel of 600 bread wheat landraces from the ICARDA's gene bank was selected for this study. This panel was a collection of landrace accessions from Syria (376), Turkey (157), Iran (47), Greece (7), Iraq (7), Spain (3), Jordan (2) and Palestine (1). The AM panel was evaluated for seedling and APR against *PstS2* and *Warrior* pathotypes. The complete list of landrace accessions and the country of origin is presented in Supplemental Table S1.

**Core Ideas**

- The bread wheat landraces preserved in ICARDA gene bank are key genetic resource for resistance to biotic stresses
- Stripe rust is one of the most important disease of wheat
- Novel genomic regions identified harboring seedling and adult plant resistance against *PstS2* and *Warrior* races of stripe rust

### 2.2 Seedling stage stripe rust assessment

The AM panel was assessed for seedling resistance against the two *Pst* pathotypes, *PstS2* and *Warrior*, which were previously collected in Turkey and characterized at the Cereal Rust Bio‐safety Laboratory (BSL) of the Regional Cereal Rust Research Center (RCRRC) in Izmir. The *PstS2* and *Warrior* races used in this study belonged to *PstS2v27* and *PstS7vWarrior* lineages and the virulence/avirulence formulae of the two races along with the differential set used in the study are given in Supplemental Tables S2 and S3. Eight to ten seeds of each accession were used in assessment of seedling resistance. Seeds were planted in 7 cm by 7 cm by 7 cm plastic pots containing a mixture of soil, compost, and sand in a ratio of 1:1:1. Seedlings were grown at 17–20 °C for 10–12 days in a spore‐proof growth chamber. Inoculation was carried out when the first leaf was fully expanded, and the second leaf was half emerged. Fresh 10 mg urediospores of each pathotype, suspended in the light mineral oil (Soltrol 170), were sprayed onto the seedlings with an atomizer. Inoculated seedlings were allowed to dry for few minutes at room temperature followed by fine misting using distilled water and placing in wet plastic cages containing water at the bottom. The seedlings were then incubated in a dark room for 24 hours at 8–10 °C with relative humidity close to 100%. The seedlings were then transferred to a growth chamber with a temperature regime of 15 °C for 16 hours light and 8 hours of dark at 10°. Disease scoring was done on all the germinated seeds which was on average 6–8 seeds per accession. Disease assessment was performed 15 days after inoculation using the 0–9 scale (McNeal, Konzak, Smith, Tate, & Russell, 1971) for seedling infection types (ITs). The range of seedling infections between 0 and 6 were considered low infection types and scores of 7, 8, and 9 were considered as high infection types. The low and high infection types were converted to 0 and 1, respectively, when the data was used for AM analysis.

### 2.3 Field assessment on adult plants

The field experiments were carried out at the RCRRC during cropping season 2016 (IZM16), 2018 (IZM18) and 2019 (IZM19). The experiment was laid out as an augmented design with un‐replicated test entries and repeated check rows in 30 blocks. Each block contained 20 test entries and two checks. Thirty seeds from each accessions were planted in a 1‐meter row with 30 cm spacing between the rows. To ensure sufficient inoculum production for disease infection, a mixture of the universally susceptible varieties ‘Morocco’, ‘Seri 82’, and ‘Avocet S’ along with the locally susceptible varieties ‘Bolani’, ‘Basribey’ (also derived from the CIMMYT cross ‘Kauz’), and ‘Cumhuriyet 75’, ‘Kunduru’, ‘Kasifbey’, and ‘Gonen’ was planted as spreader after every 20 rows, as well as spreader rows bordering the nurseries. The experiments were managed as per the standard local agronomic practices during the crop season. Genetic and residual variances were estimated for the trials using the software Genstat (http://Genstat.co.uk). Residual (or Restricted) Maximum Likelihood (REML) procedure (Patterson & Thompson, 1971) method was used for estimating variance components where random model is Block + Geno. We used the following VFUNCTION in Genstat to calculate functions of variance components from REML analysis.

$$\begin{array}{c} \mathrm{VFUNCTION}\left[\mathrm{RANDOM}=\mathrm{BLOCK}+\mathrm{GENO}\right]\\ \;\mathrm{NUMERATOR}=!\;\left(0,1,0\right);\\ \;\mathrm{DENOMINATOR}=!\left(0,1,1\right);\\ \;\mathrm{FUNCTIONVALUE}=\mathrm{HeritAll};\;\mathrm{SE}=\mathrm{SeHeritAll}. \end{array}$$

A measure of broad‐sense heritability for yellow rust is the ratio of genetic variance ($\sigma_{g}^{2}$) to phenotypic variance ($\sigma_{g}^{2}+\sigma_{\varepsilon }^{2}$) and is represented as follow:

$$\;h^{2}=\frac{\sigma_{g}^{2}}{\sigma_{g\;+\;}^{2}\sigma_{\varepsilon \;}^{2}}\;$$

*PstS2* and *Warrior (PstS7)* pathotypes collected from previous years and preserved at RCRRC were multiplied at the BSL using susceptible variety Avocet S. Freshly collected urediniospores were used for field inoculations. The AM panel along with the spreader rows bordering the experiment were artificially sprayed with a mixture of the two races in talcum powder using a backpack sprayer at seedling, tillering and booting stages. The field was irrigated through a mist irrigation system.

Field scoring started when disease severity reached 100% on the susceptible checks, ‘Morocco’ and ‘Avocet S’. Because of conducive environmental conditions during January–February, the onset of the stripe rust (under artificial inoculation) usually starts at early February and reach to full disease severity in susceptible genotypes by mid‐February in which the plants are usually at tillering stage. This is a unique condition for evaluation of resistance in wheat germplasms at regional rust phenotyping platform in Izmir. Adult‐plant responses were recorded three times at 10‐day intervals for the major infection types R, MR, MS, and S (Roelfs et al., 1992), and the disease severities (0–100%) following the Modified Cobb's Scale (Peterson, Campbell, & Hannah, 1948). All three recordings were averaged and the Coefficients of Infection (CI) were calculated for infection types and disease severities following Saari and Wilcoxson (1974).

### 2.4 DNA extraction and genotyping

Genomic DNA was extracted from fresh leaves were collected from three individual 10‐day old seedlings using a modified CTAB (cetyltrimethylammonium bromide) method (Hoisington, Khairallah, & González de León, 1994). The seedling leaves were collected in labeled Eppendorf tubes and stored in an Ultra freezer at −80 °C for subsequent DNA extraction. Leaf samples were ground using a tissue lyser (Tissue Lyser II from QIAGEN) untill a fine powder was obtained; 0.1 g of the powdered leaf samples were used for DNA extraction using the CTAB method (Doyle, 1990). The extracted DNA was dissolved in 100 μl tris‐EDTA (TE) buffer. The samples were analyzed on 1% agarose gel for the purity test and quantified with a spectrophotometer (NanoDrop ND1000). The DNA samples were then kept at −80 °C.

A high‐throughput genotyping by sequencing (GBS) method called DArTseq technology (Sansaloni et al., 2011) was used for all samples at the Genetic Analysis Service for Agriculture (SAGA) at the International Maize and Wheat Improvement Center (CIMMYT) in Mexico, and supported by the CGIAR Research Program (Sansaloni et al., 2011).

### 2.5 Population structure, linkage disequilibrium (LD) and kinship analysis

To analyze the genetic variation within the population, 1072 unlinked SNP markers were used using STRUCTURE software (v.2.3.4), which implements a model‐based Bayesian cluster analysis (Pritchard, Stephens, & Donnelly, 2000). The program was operated for ten independent runs for robust evaluation with the putative number of sub‐populations ranging from k = 1–10, assessed with a burn‐in period of 50,000 steps followed by 50,000 recorded Markov‐Chain iterations using an admixture model. The best K value representing the optimum number of clusters in the populations was estimated as Delta K (ΔK) based on the rate of change in the log probability of data between successive values using Structure Harvester, as described by Evanno et al. (2005). Structure analysis was performed multiple times with altering parameters and iterations to reduce clustering error. Population structure was also analyzed by Principal Component Analysis (PCA) using the R software package (R Core Team, 2013), PCAdapt, which implements the method described by Luu, Bazin, and Blum (2017). The LD among the markers was estimated for the AM panel in the program TASSEL (v.5.2.24) (Bradbury et al., 2007), using the observed vs. expected allele frequencies. The LD decay was measured as the distance at which the average *r^2^* between pairwise SNPs dropped to half of its starting maximum average value (Huang et al., 2010). TASSEL (v.5.2.24) was used to derive the population kinship matrix based on the scaled IBS (identity by state) method, using the complete set of markers that passed quality filtering, as reported by Gao, Turner, Chao, Kolmer, and Anderson (2016).

### 2.6 Association mapping analysis

A total of 152K SNPs were discovered de novo. After eliminating the SNP markers call rate of less than 0.8, minor allele frequencies of less than 0.05 (MAF < 0.05), and maximum missing counts of 20%, a set of 25,169 high‐quality SNP markers were used in the association analysis. The genetic framework of the polymorphic SNPs was constructed using BLAST alignment of each allele sequence with a reference genome of the Chinese Spring IWGSC RefSeq v1.0 assembly (Appels et al., 2018). Since both model‐based Bayesian and PCA revealed a population structure in the panel, marker trait association was carried out based on the Mixed Linear Model (MLM Q+K), where, Q is the first three principal components as covariates and K is the kinship matrix, using the Genome Association and Prediction Integrated Tool (GAPIT) (Lipka et al., 2012) under the open‐source R environment. Significant markers were identified based on estimating False Discovery rate (FDR) values for each experiment (Benjamini & Hochberg, 1995; Ozkuru et al., 2019). Markers with a minimum threshold of experiment‐wise FDR (q) ≤ 0.05 were considered significant. A previously developed integrated map (Bulli et al., 2016) was used to determine the relationships of the SNPs identified in this study with previously reported *Yr* genes and QTL. Names assigned to the novel QTL identified in this study start with the prefix “*Q*” for QTL, followed by “*Yr*” for yellow rust, chromosome name, and “*seedling*” for seedling trait, “APR” for adult plant resistance.

## 3 RESULTS

### 3.1 Seedling stage phenotypic response

Since the seedling assessment was performed to identify possible race‐specific seedling resistance genes and therefore on a 0 to 9 scale, ITs of 0 to 6 were considered as an indication of incompatible resistance responses (low IT), and the ITs of 7 to 9 as compatible resistance responses (high IT), which were converted to 0 and 1 for low and high ITs, respectively.

In the seedling test of the landraces against of *PstS2* pathotype, 22.7% of the accessions were resistant (low IT), 71.17% were susceptible (high IT). Of 376 accessions of the Syrian origin, 283 accessions (75%) were susceptible. In total 110 (70%) and 27 (57%) landraces collected from Turkey and Iran, respectively, showed susceptible response. The three accessions from Jordan and Palestine were susceptible, while the three accessions from Spain and the seven accessions from Greece all showed a resistant reaction.

In the case of the *Warrior* pathotype, a similar trend was observed with 80% of all the accessions showing a varying degree of susceptible response, and only 16% showed resistant reaction types. From the tested accessions against *Warrior* pathotype, 80% and 83.5% of the accessions from Iran and Syria showed susceptible responses, respectively, while 73% of the landraces collected from Turkey showed susceptible reactions. The three accessions from Jordan and Palestine showed susceptible responses, while the seven landraces from Greece and the three from Spain showed resistant response. The frequency of resistant and susceptible genotypes according to their country of origin are presented in Table 1. Minimum, maximum and mean scores for seedling and adult plant data are given in Table 2.

**TABLE 1:** Seedling response of 600 bread wheat landraces against *PstS2* and *Warrior* pathotypes

	Pathotypes					
	*PstS2*			*Warrior*		
Country	Resistant	Susceptible	No data	Resistant	Susceptible	No data
Syria	76	283	17	48	314	14
Turkey	32	110	15	35	116	6
Iran	18	27	2	9	38	0
Iraq	2	3	2	1	5	1
Greece	5	1	1	2	4	1
Palestine	0	1	0	0	1	0
Jordan	0	2	0	0	2	0
Spain	3	0	0	2	1	0

**TABLE 2:** Basic statistics of seedling and adult plant response of bread wheat land races against *PstS2* and *Warrior* pathotypes of stripe rust, estimates of variance components and broad‐sense heritability

Parameter	Seedling *PstS2*	Seedling *Warrior*	IZM16	IZM18	IZM19	Combined
Minimum	1.0	1.0	2.0	1.0	1.0	1.0
Mean	6.8	7.2	29.7	31.8	26.0	29.1
Maximum	9.0	9.0	90.0	100.0	100.0	100.0
σ^2^_G_			591.5**	1048.1**	788.1**	178.83**
σ^2^_E_			32	20.1	22.8	17
σ^2^_P_			623.5	1068.2	810.9	195.83
Heritability			0.95	0.98	0.97	0.91

^*^Significance at the 5% probability level.

^**^Significance at the 1% probability level.

σ^2^_G_ = estimates of genotypic variance.

σ^2^_E_ = estimates of environmental variance.

σ^2^_P_ = estimates of phenotypic variance.

### 3.2 Field assessment of resistance

In adult‐plant assessment, the estimates of genetic variance identified significant differences among the landraces (Table 2). Although a small variation was observed in the diseases severity scores of the tested accessions during the three years, the overall field infection type patterns of the 600 landraces were consistent over the years. During the 2016 trial, 280 (6.6%) of accessions showed a resistance response (CI = 0 to 19), 89 (14.8%) exhibited moderate resistance (CI = 20 to 39), 105 (17.5%) showed a moderately susceptible response (CI = 40 to 59), and 122 (20.3%) of the accessions were susceptible (CI = 60 to 100). During the year 2018, 310 (51.6%) of the genotypes showed a resistance response, 76 (12.6%) of the genotypes showed moderately resistant response, 52 (8.6%) exhibited a moderately susceptible response, and 137 (22.8%) of the accessions were susceptible. In the year 2019, 346 (57.6%) of the genotypes showed a resistant response, 13.5% showed a moderately resistant response, while 12.6% exhibited a moderately susceptible reaction, whereas 85 (14%) of the genotypes were susceptible.

**FIGURE 1:**
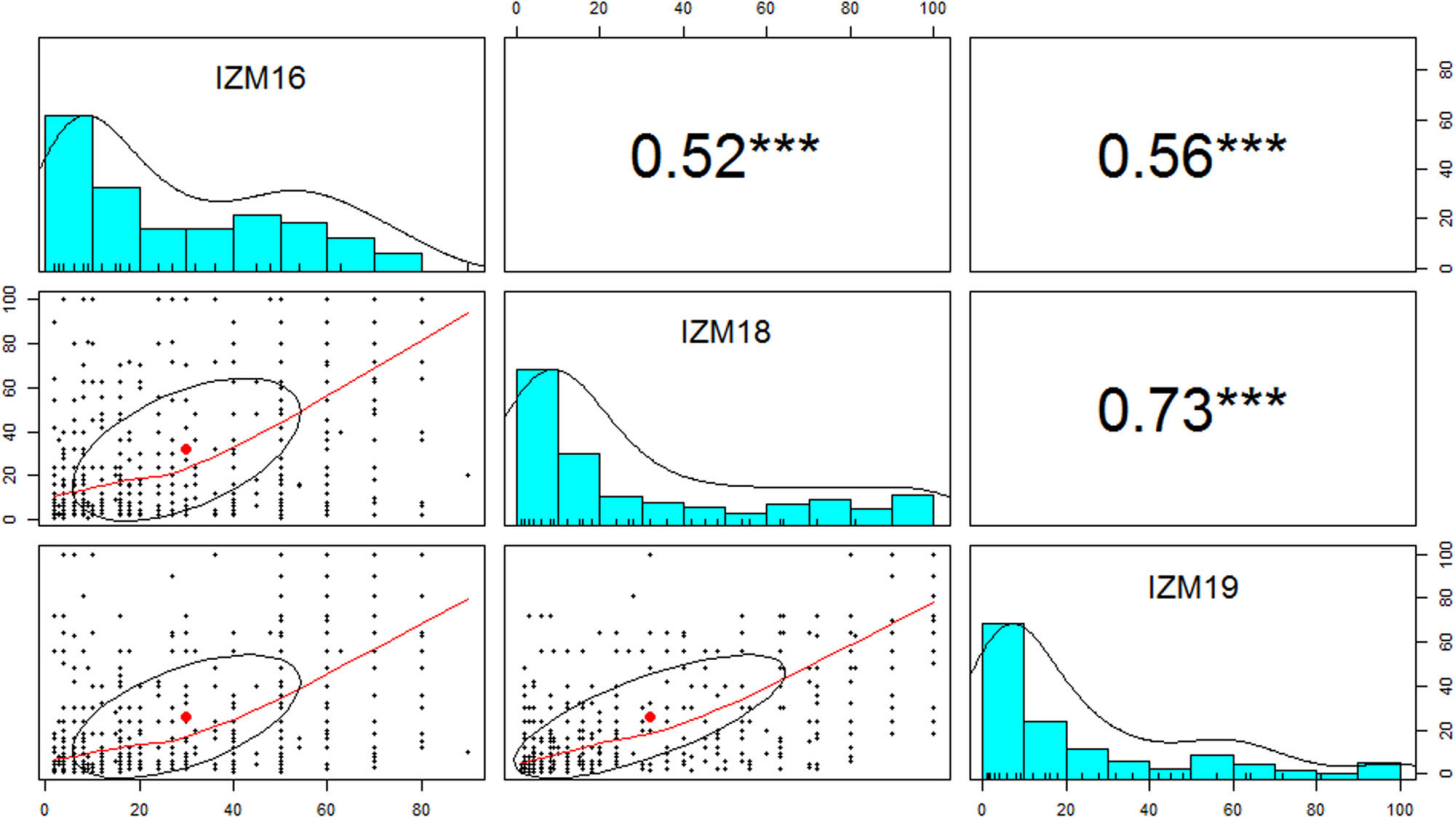
Scatter plot, histogram and pearson correlation analysis of 600 bread wheat landraces in field condition over three years. The X and Y axis represents stripe rust coefficient of infection (CI)

Over the three years, 71.8% of the accessions showed resistant to moderate resistant response whereas 28. 7% accessions showed moderate susceptible to susceptible response in the field. The year‐wise frequency of disease response of the genotypes according to their geographical origins and correlation amongst the years is presented in Figure 1 and Table 3.

**TABLE 3:** Field response of 600 bread wheat landraces

			Adult‐plant response			
	Izmir 2016		Izmir 2018		Izmir 2019	
Country	R ‐ MR	MS‐S	R ‐ MR	MS‐S	R ‐ MR	MS‐S
Syria	246	126	180	131	172	108
Turkey	118	39	125	28	135	17
Iran	29	18	32	14	32	13
Iraq	3	4	3	4	2	5
Greece	4	3	6	1	6	1
Palestine	0	1	1	0	0	1
Jordan	0	2	1	1	1	1
Spain	2	1	3	0	2	0

### 3.3 Analysis of SNP markers and LD

**FIGURE 2:**
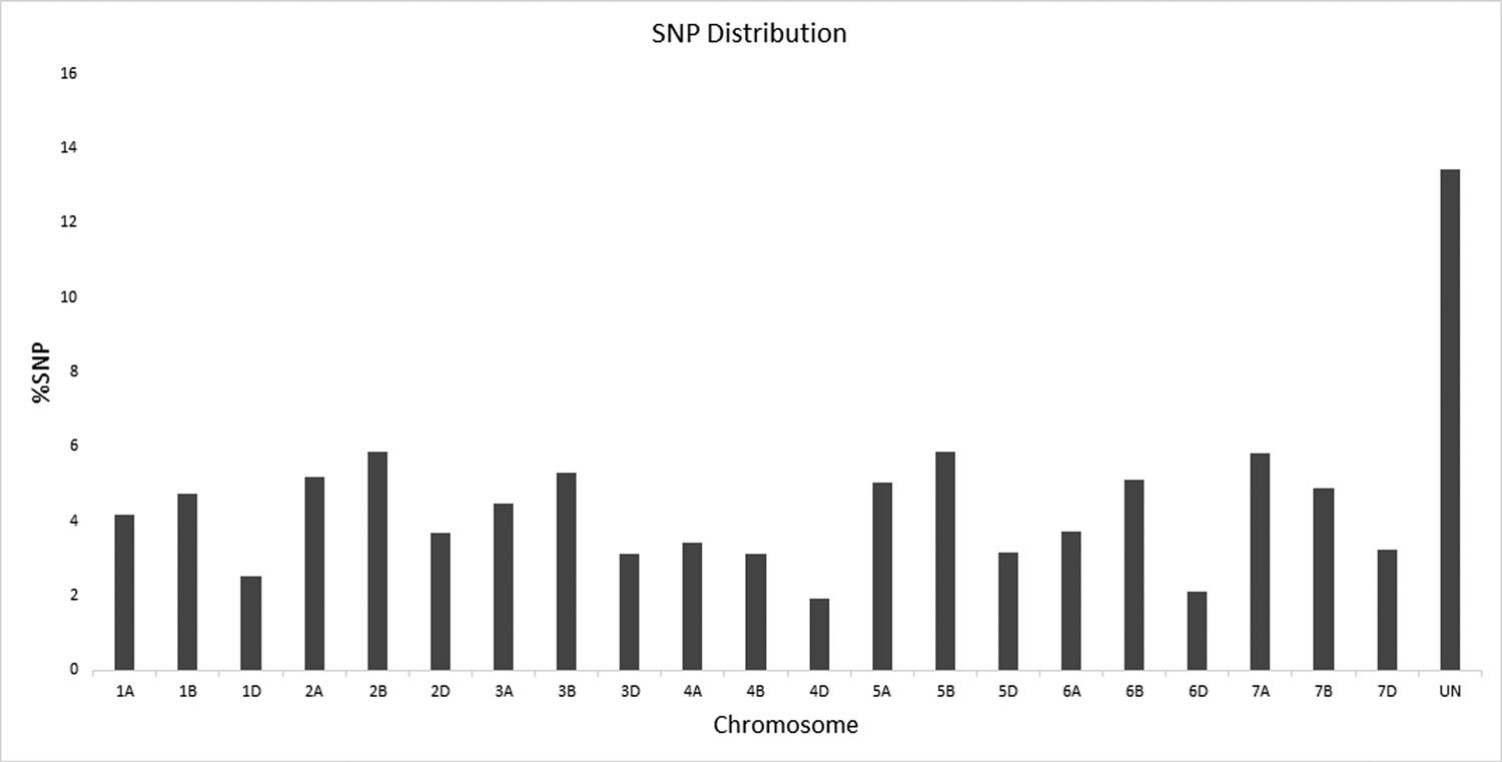
Distribution of single nucleotide polymorphisms (SNPs) on the chromosomes

A set of 25,169 high‐quality SNP markers was used for association analysis. Out of these 21,789 markers were distributed across all 21 chromosomes with an average of 1,000 markers per chromosome; 3,380 (13%) of the markers were not assigned to any chromosomal location. The maximum marker density was observed on chromosome 2B with 1,477 SNPs, and chromosome 4D showed a minimum markers density with 487 SNPs (Figure 2). The marker density for the A and B genomes was almost similar with an average of 1,145 and 1,255 markers per chromosome, respectively. However, the D genome had a relatively poor marker density with an average chromosome coverage of 711 markers. The extent of LD was estimated for the diversity panel using TASSEL software (Bradbury et al., 2007). It indicated the B sub‐genome to have the highest LD, followed by the A and D genomes, respectively (Supplemental Figure S1). LD decreased with the increase in physical distance between the marker loci. Average LD decay was observed after 0.4 Mb in A genome, after 0.5 Mb in B genome, and after 0.3 Mb in D genome.

### 3.4 Population structure analysis

Out of the 600 bread wheat landraces, 88% (533) were collected from Syria and Turkey alone, while 7% (47) were derived from Iran, and the rest 3% (20) are from Iraq, Spain, Greece, Jordan, and Palestine. The results indicated that the population structure was best represented at K = 2. In the plot of K against ΔK, there was a slope after K = 2 following the flattening of the curve. Population structure did not correspond to grouping based on the origin of the landraces. However the landraces could be divided into two major groups mostly from Syria and Turkey (Figure 3). The scree plot from PCA identified three clusters as the line levelled off at PC = 3 when plotted against PC = 1 to 30. It has been reported that the STRUCTURE software does not provide an optimal K when genetic structure of individuals is complex and unrelated (Zegeye, Rasheed, Makdis, Badebo, & Ogbonnaya, 2014). Therefore, the population was considered to be divided into three clusters with admixture (Figure 3).

**FIGURE 3a:**
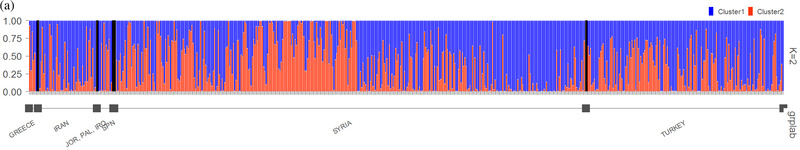
Population structure results at K = 2. Every single line represents an individual land race

**FIGURE 3b:**
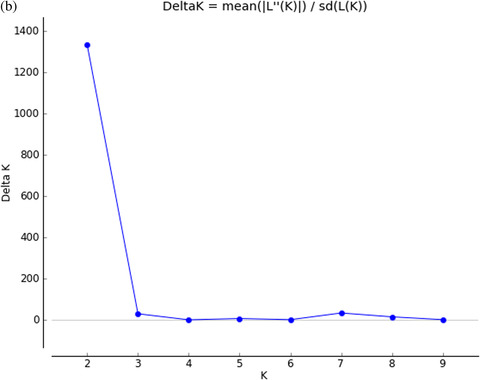
The plot of the scaled average logarithm of the probability of data likelihood [LnP(D)] and Delta K (ΔK) with K allowed to range from 2 to 10

**FIGURE 3c:**
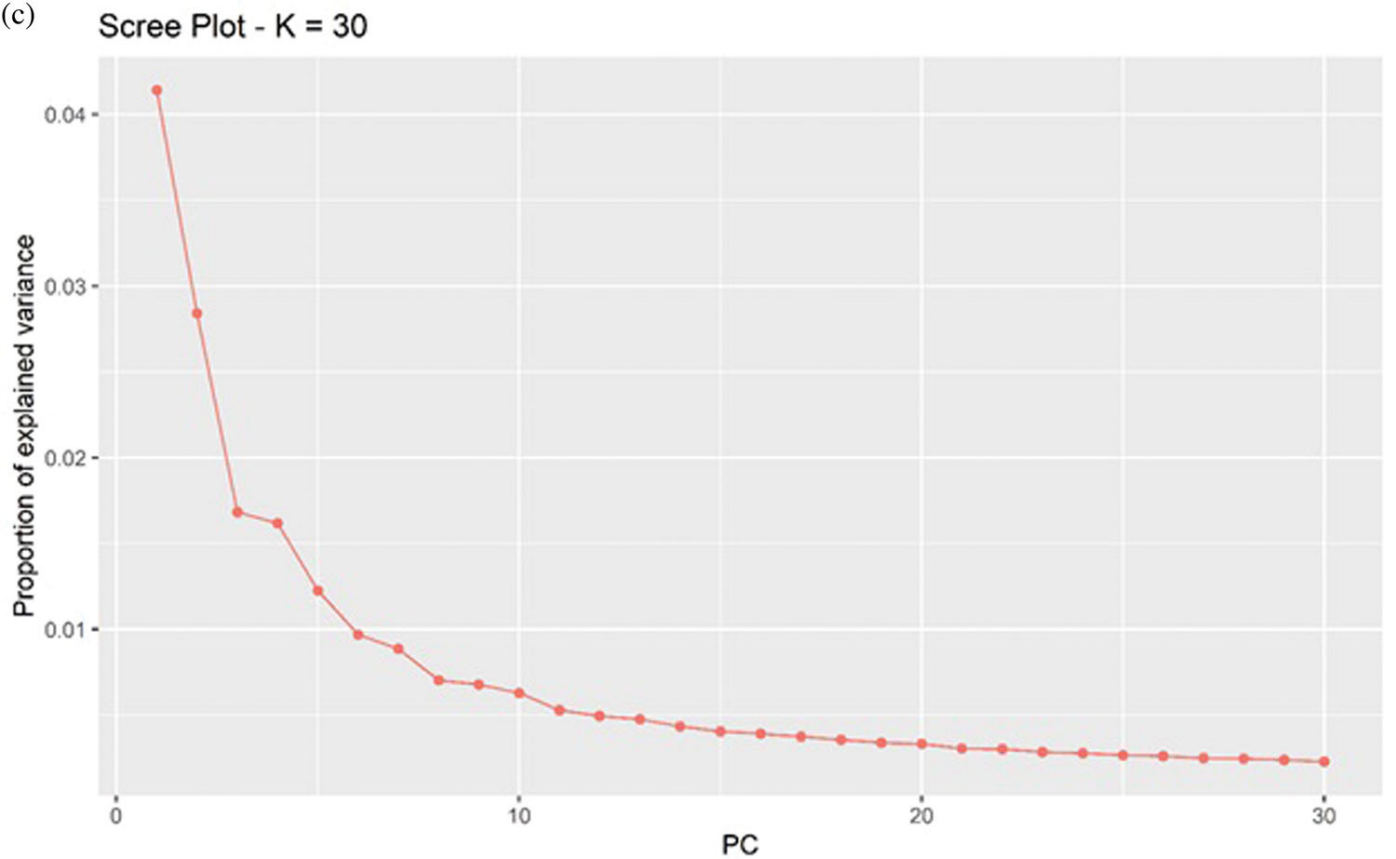
Scree plot derived from PCA with K values ranging from 1 to 30

**FIGURE 3d:**
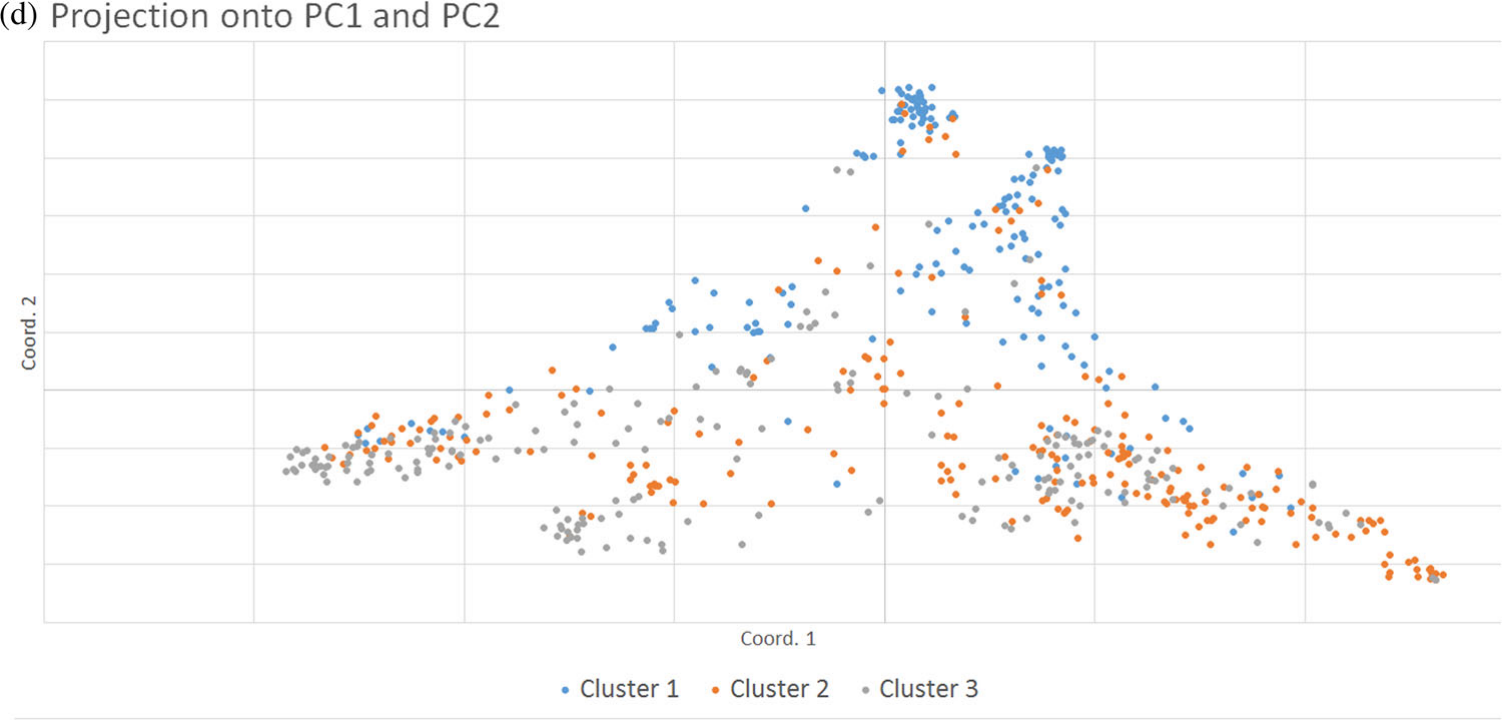
First two principle components of PCA showing different population clusters

### 3.5 Association mapping analysis

**FIGURE 4:**
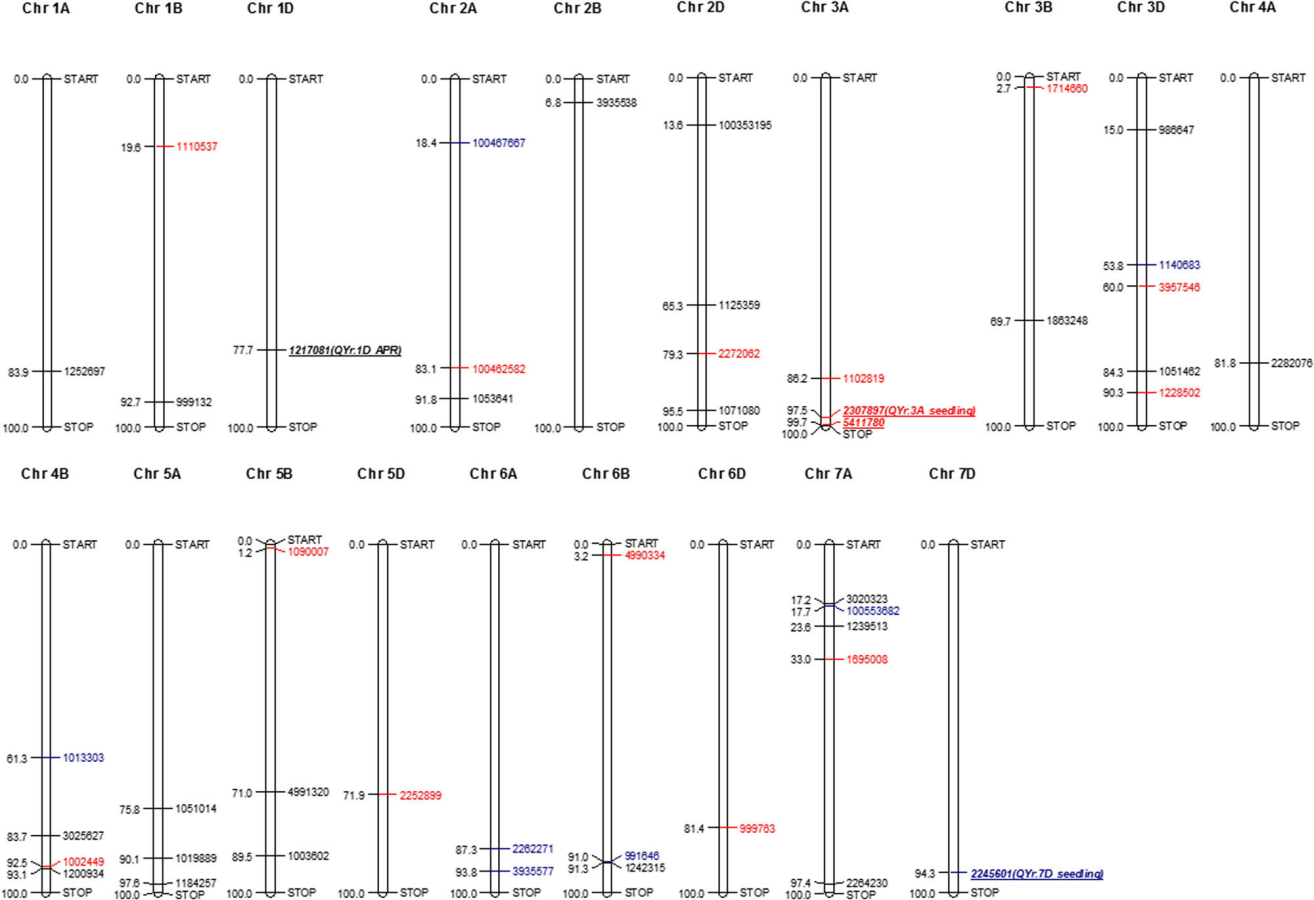
Significant single nucleotide polymorphisms (SNPs) detected in the study based on the wheat consensus genetic map (Bulli et al., 2016)

Out of the 25,169 high‐quality SNP markers, 21,789 (86.6%) were of known positions on the reference genome (Appels et al., 2018), of which 8,020, 8,787 and 4,982 were specific to A, B, and D genomes, respectively. A total of 47 SNP markers from 19 chromosomes were significantly associated with resistance to stripe rust at the adult plant and seedling stage (FDR ≤ 0.05) (Table 4; Table 5). The highest number of SNP markers were identified on chromosomes 3D and 7A, i.e. 5, followed by 2D, 4B, and 5B with 4 SNPs on each of the chromosomes. The A genome had the highest number of significant SNPs, i.e. 18, followed by the B genome with 17 SNPs. and the D genome with 13 SNPs. For seedling stage resistance, 8 SNPs on seven chromosomes (2A, 3D, 4B, 6A, 6B, 7A, and 7D) were identified to be associated with seedling stage resistance against the *PstS2* pathotype. Seedling stage analysis for the *Warrior* pathotype identified 15 significant SNP markers on 12 chromosomes (1B, 2A, 2D, 3A, 3B, 3D, 4B, 5B, 5D, 6B, 6D and 7A) to be associated to resistance at the seedling stage (Table 4). All‐stage resistance QTL were detected on chromosomes 3D, 4B, 6B, and 7A. Of the 47 significant SNP markers, 24 SNPs on 14 chromosomes were associated with resistance to stripe rust at the adult plant stage (Table 5). In addition, several SNPs were found significantly associated with both adult plant and seedling stage resistance expression, but with unknown chromosomal positions. Therefore, these could not be co‐localized with any of the previously identified *Yr* genes/QTL (Supplemental Table S4). Four SNPs on three genomic regions were mapped far from any previously identified *Yr* gene/QTL. Hence, these three genomic regions most likely tag new *Pst* resistance loci (Figure 4). The remaining genomic regions putative to field and seedling resistance were mapped close to known *Yr* genes and QTL.

**TABLE 4:** Genomic regions significantly associated with seedling resistance to stripe rust in 600 bread wheat landraces collection from ICARDA gene bank

**SNP** ^a^	**Trait**	**Chr**	**Position (bp)** ^b^	**‐log10p** ^c^	**R²**	**Previously mapped *Yr* gene/QTL** ^d^
1110537	Warrior	1B	1.35 × 10^8^	3.52^*^	0.047	*Yr15, YrCH52, Yr64, YrAlp*
100467667	PstS2	2A	1.44 × 10^8^	3.64^*^	0.074	*Yrxy2*, QTLs QYr.ucw‐2A.3, QYr.sun‐2AS_Kukri
100462582	Warrior	2A	6.49 × 10^8^	3.47^*^	0.047	*QYr.inra_2AL.2_CampRemy*
2272062	Warrior	2D	5.17 × 10^8^	3.36^*^	0.046	*QYr.tam_2D_Quaiu, QYr.jic‐2D_Briagdier*
2307897	Warrior	3A	7.32 × 10^8^	3.71^*^	0.049	*QYr.3A_seedling*
5411780	Warrior	3A	7.49 × 10^8^	3.36^*^	0.046	*QYr.3A_seedling*
1102819	Warrior	3A	6.47 × 10^8^	3.64^*^	0.048	*QYrdr.wgp‐3AL (IWA6834), QYr.wsu‐3A (IWA3401)*
1714660	Warrior	3B	23162218	3.59^*^	0.048	*wPt‐800213‐IWA6510*
1140683	PstS2	3D	3.31 × 10^8^	3.37^*^	0.072	*Yr45*
1228502	Warrior	3D	5.56 × 10^8^	3.62^*^	0.048	*IWA3012_seedling*
3957546	Warrior	3D	3.69 × 10^8^	3.37^*^	0.046	*Yr45*
1013303	PstS2	4B	4.14 × 10^8^	3.51^*^	0.073	*Yr50, QYr.sun‐4B_Janz*
1002449	Warrior	4B	6.23 × 10^8^	3.91^**^	0.05	*QYr.wpg‐4B.2 (IWA3994)*
1090007	Warrior	5B	9075119	4.09^**^	0.052	*Yr47*
2252899	Warrior	5D	4.07 × 10^8^	4.13^**^	0.052	*Yr.caas‐5DS_Jingshuang 16*
3935577	PstS2	6A	5.8 × 10^8^	4.1^**^	0.077	*QYr.cim‐6AL_Francolin*
2262271	PstS2	6A	5.4 × 10^8^	3.33^*^	0.071	*QYr.caas‐6AL (IWB39473) Zhong 892*
991646	PstS2	6B	6.57 × 10^8^	3.72^*^	0.074	QYr.ucw‐6B (IWA7257)
4990334	Warrior	6B	23343483	3.39^*^	0.046	*Yr35*, QYr.ufs‐6B_Kariega
999763	Warrior	6D	3.86 × 10^8^	4.82^**^	0.053	*QYr.ufs‐6D_Cappelle‐Desprez*, IWA4455_*Seedling*
100553682	PstS2	7A	1.31 × 10^8^	4.09^**^	0.077	*Yr61, QYr.caas‐7A_Jingshuan16*
1695008	Warrior	7A	2.44 × 10^8^	3.76^*^	0.049	*Yr61, QYr.sun‐7A_CPI133872*
2245601	PstS2	7D	6.02 × 10^8^	3.56^*^	0.073	*QYr.7D_seedling*

^a^ SNP index from the GBS data.

^b^ Physical positions based on the IWGSC RefSeq. 1.0.

^c^ Significance. Experiment‐wise (Benjamini‐Hochberg FDR adjusted):

^*^ *FDR (q) ≤ 0.05*, ^**^ *FDR (q) ≤ 0.01*.

^d^ References are given in the text.

**TABLE 5:** Genomic regions significantly associated with field‐based adult plant resistance (APR) to stripe rust in 600 bread wheat landraces collection from the ICARDA gene bank

SNP^a^	Chr	Position (bp)^b^	−log10p ^c^	R²	Trait	Previously mapped *Yr* gene/QTL^d^
1252697	1A	498822949	3.43^*^	0.29	IZM16	*QYr.sun‐1A_Janz*
999132	1B	639941356	4.35^**^	0.27	IZM19	*Yr29/Lr46, QYr.jic‐1B_Guardian*
100495600	1B	637938403	3.33^*^	0.26	IZM19	*Yr29/Lr46, QYr.jic‐1B_Guardian*
1217081	1D	385129089	3.59^*^	0.29	IZM16	*QYr.1D_APR*
1053641	2A	717007485	4.02^**^	0.29	IZM16, IZM18	*Yr1, QYr.inra_2AL.2_CampRemy*
3935538	2B	54545198	3.76^*^	0.26	IZM19	*wPt‐6271, QYr.inra‐2BS_Renan*
1071080	2D	622918234	3.40^*^	0.26	IZM19	*Yr54*
1125359	2D	425739911	3.32^*^	0.26	IZM18	*Yr55, QYr.tam_2D_Quaiu*
100353195	2D	89091110	3.30^*^	0.29	IZM16	*QYr.caas‐2DS_Libellulaz, QYr.wpg‐2D.1 (IWA1939)*
1863248	3B	579446373	3.83^*^	0.29	IZM16	*QYr.cim‐3B_Pastor, QRYr3B.2*
1051462	3D	519174887	4.16^**^	0.29	IZM16	*Yr45, IWA3012_Seedling*
986647	3D	92367815	3.67^*^	0.26	IZM18	*QYr.tam‐3D_Quaiu*
2282076	4A	609314981	3.99^**^	0.26	IZM19	*QYr.sgi‐4A.1_Kariega, QYr.sgi‐4A.2_Kariega*
3025627	4B	564382746	4.11^**^	0.27	IZM16, IZM18	*Yr50, QYr.wpg‐4B.2 (IWA3994)*
1200934	4B	627519990	3.67^*^	0.26	IZM18	*Yr50, QYr.wpg‐4B.2 (IWA3994)*
1019889	5A	639997788	3.56^*^	0.26	IZM18	*QYr‐5A_Opata85, QYr.cim‐5AL_Pastor*
1051014	5A	538581590	3.45^*^	0.26	IZM19	*IWA6949_APR*
1184257	5A	693326861	3.38^*^	0.29	IZM16	*Yr48*, QYr.ucw‐5A.1, QYr.ucw‐5AL_PI610750
1003602	5B	638408729	4.12^**^	0.26	IZM19	*QYr.sun‐5B_Wollari, QTr.ui‐5B_IDO444, QYrdr.wgp‐5BL.1 (IWA6271)*
4991320	5B	506794189	3.30^*^	0.29	IZM16	*QYr.caas‐5BL.2_Libellula,QYr.tem‐5B.2_Flinor, QYr.inra‐5BL.2_Camp Remy*
1242315	6B	658625197	3.55^*^	0.26	IZM18	*QYr.ucw‐6B* (IWA7257)
3020323	7A	126997677	4.61^**^	0.27	IZM18	*Yr61, QYr.caas‐7A_Jingshuan16, QYr.sun‐7A_CPI133872*
2264230	7A	718149527	3.38^*^	0.26	IZM19	*QYr.sgi‐7A_Kariega*
1239513	7A	174198127	3.36^*^	0.26	IZM18	*Yr61, QYr.caas‐7A_Jingshuan16, QYr.sun‐7A_CPI133872*

^a^ SNP index from the GBS data.

^b^ Physical positions based on the IWGSC RefSeq. 1.0.

^c^ Significance. Experiment‐wise (Benjamini‐Hochberg FDR adjusted):

^*^ *FDR (q) ≤ 0.05*, ^**^ *FDR (q) ≤ 0.01*.

^d^ References are given in the text.

### 3.6 Effect and distribution of resistance/favorable alleles

**FIGURE 5:**
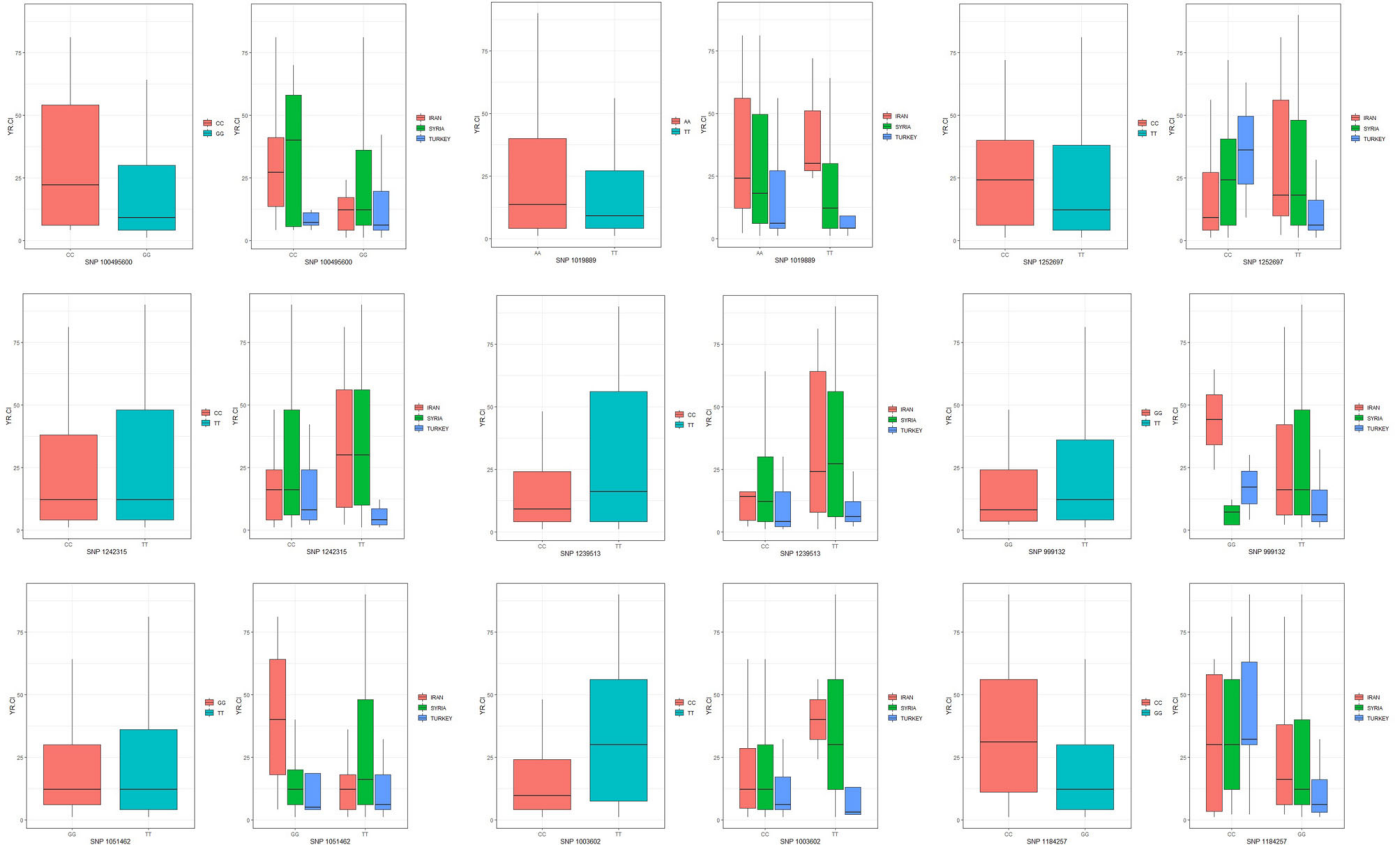
Boxplots of single nucleotide polymorphisms (SNPs) associated with *Pst* resistance in the AM panel

Landraces from Syria, Iran and Turkey were used to identify the favorable alleles of the significant SNPs associated to *Pst* resistance, as 96% of all the accessions originated from these three regions. Resistance alleles of the significantly associated SNPs showed a difference in *Pst* response of up to 30% with the unfavorable alleles. There appeared to have been no single type of selection pressure, as the resistance alleles were distributed randomly across the geographical regions. Furthermore, in some cases favorable alleles of the significant SNPs in the whole population differed within the geographic regions (Figure 5). In general, as is evident from the population structure, the landraces from Syria and Turkey differ in the resistance alleles of the significant SNPs. In case of Iran, however, with some exceptions mostly the resistant alleles were the same as in the Syrian landraces, revealing the relatedness between Iranian and Syrian landraces.

## 4 DISCUSSION

There is an increased use of wheat landraces for the enhancement of genetic diversity and the mining of desirable genes (Yao et al., 2019). Therefore, wheat landraces are considered a key genetic resource for modern wheat breeding (Sehgal et al., 2016).

### 4.1 Phenotypic variability, population structure and LD

The AM panel showed 46.6%, 51.6% and 57.6% adult plant resistance in the three years of field testing, respectively, which is similar to the stripe rust response in Izmir to Turkish landraces reported by Sehgal et al. (2016). The Iranian and Syrian landraces were considerably similar in terms of genetic resistance. The identification of favorable alleles within populations is important for potential use of the material as donor parents in breeding programs. The number of population genetic clusters found in our study is similar to previous GWAS studies (Bulli et al., 2016; Chen et al., 2019; Kertho et al., 2015; Ozkuru et al., 2019). However, the population structure identified in this study had lesser number of subpopulations than many previously reported GWAS studies (Jighly et al., 2015; Liu et al., 2017; Zegeye et al., 2014). This may be due to the geographic proximity of the landraces collected for this study. Regions in Turkey and Syria are part of the Fertile Crescent, which is considered as the center of origin and diversity of wheat, where wheat and its relatives have been cultivated for millennia (Bird, 1999). Local wheat landraces would have evolved over time by being both adaptive to the local ecology, as well as resistant to prevalent biotic and abiotic stresses (Akcura, Kadir, & Hocaoglu, 2017). The advantage of these landraces over commercial varieties as potential resistance gene donors is their cultivation over many years by local farmers, leading them to adapt to changing climatic conditions and evolving disease resistance that could be expected to be more durable and novel (Sehgal et al., 2016). An earlier example of the potential value of millennia of co‐evolution of host and pathogen is the durability of the transfer of common bunt resistance originating from Turkish landraces to modern cultivars, saving millions of dollars for the wheat industry (Bonman et al., 2006).

Approximately 30% of all the landraces from Syria and Turkey consistently showed APR in all three years, revealing high genetic diversity in both the clusters in the population. In this study, nine landraces from Turkey were resistant at both the seedling and adult plant stage against both *PstS2* and *Warrior* pathotypes of the stripe rust, revealing high genetic variation and usefulness of Turkish wheat landraces for stripe rust resistance. The Turkish national wheat breeding program can effectively utilize landraces for resistance to biotic stresses, tolerance to abiotic stresses, and end‐use quality improvement (Akcura, 2011; Karagoz, 2014; Morgounov et al., 2016).

LD is reported to decay rapidly in outcrossing species, such as maize, and slower in self‐pollinated crops, such as wheat (Dinesh et al., 2016; Yu, Deng, Xiang, & Tian, 2014). The LD decay extension depends upon the genetic and physical distances in the genome, and indicates the mapping resolution in the panel and the number of markers required for successful AM (Mather et al., 2007; Vos et al., 2017). LD decay varies significantly amongst different wheat populations (Chao, Zhang, Dubcovsky, & Sorrells, 2007). In wheat LD is reported to decay within 10 cM (Bulli et al., 2016; Chen et al., 2019; Maccaferri et al., 2015; Muleta et al., 2017; Sehgal et al., 2016; Zegeye et al., 2014), within 20 cM (Benson, Brown‐Guedira, Paul Murphy, & Sneller, 2012; Crossa et al., 2007), or at about 5 × 10^7^ bps (Juliana et al., 2018). Sajjad et al. (2014) reported an LD decay of 80 cM on chromosome 3A in spring wheat accessions using SSR markers. These previous reports indicate that LD decay varies in hexaploid wheat depending on populations and genomic regions. In this study, the LD decayed at 0.4 Mb, 0.5 Mb and 0.3 Mb for A, B and D genomes, respectively suggesting a high genetic diversity of the AM panel. Some previous studies (Chao et al., 2007; Gao et al., 2016) have reported a slower LD decay in the D genome. In the current study, we observed a faster LD decay in the D genome, compared to the A and B genomes, although the exact reason for this faster LD decay in the D genome is unknown in this AM panel. This requires further investigation, but could be attributed to natural crossing‐over events in the landraces, driving more recombination in the D genome by chance. In addition, several previous studies have also reported a faster LD decay in the D genome compared to A and B genomes in different AM populations (Ain et al., 2015; Singh et al., 2019).

### 4.2 Alignment of identified QTL to the previously mapped identified *Yr* genes/QTL

The integrated map constructed by Bulli et al. (2016) was used to compare the significant SNPs detected in the study with previously published *Yr* genes and QTL. Three genomic regions that were identified to be significant at experiment‐wise FDR ≤ 0.05, SNP1217081, SNP2307897 and SNP2245601 on chromosomes 1D, 3A, and 7D, respectively, were located in the regions where no *Yr* gene or QTL had been mapped. Therefore, these are likely tagging new stripe rust resistance loci. A total of 47 SNPs were significantly (FDR (q) ≤ 0.05) associated with stripe rust resistance in the seedling and field experiments. However, only the relationship of the 13 high‐confidence (experiment‐wise FDR (q) ≤ 0.01) SNPs across 10 chromosomes with previously mapped *Yr* genes/QTL are discussed below and the remaining are presented in Supplemental File S1.

#### 4.2.1 Chromosome 1B

SNP999132 and SNP100495600 detected in this study overlapped with the previously identified *Yr29* gene and a QTL (Melichar, Berry, Newell, MacCormack, & Boyd, 2008; William, Singh, Huerta‐Espino, Islas, & Hoisington, 2003). A variety Lalbahadur/Pavon 1BL harboring *Yr29*, which was used in the field experiments, showed a resistant to moderate susceptible reaction response. *Yr29* harbors partial resistance showing a slow rusting phenotype (Singh et al., 2001). Several studies have shown different phenotypic responses for *Yr29* (Lillemo et al., 2008; Rosewarne et al., 2006; William et al., 2003). Furthermore, *Yr29* is likely a pleiotropic gene in association with leaf rust gene *Lr46* and stem rust gene *Sr58*, hence providing multiple rust disease resistance. Based on the genetic positions of the markers, it appears that SNP999132 and SNP100495600 are likely associated with the *Yr29*/*Lr46*/*Sr58* complex.

#### 4.2.2 Chromosome 2A

SNP1053641 and SNP4008260 (Supplemental Table S4) were identified on chromosome 2A, which carries several *Yr* resistant genes. Both SNPs are identified in the same region as *QYr.inra_2AL.2_CampRemy* (Boukhatem, Baret, Mingeot, & Jacquemin, 2002) on the long arm of chromosome 2A. In the light of the virulence/avirulence formula of both races, it is observed that chromosome 2A is strongly associated with resistance to the *PstS2* race, as the *Warrior* pathotype is virulent on *Yr1*, *Yr17*, and *Yr32*. Based on the consensus genetic map (Bulli et al., 2016) and pathotype testing on our AM panel SNP1053641 and SNP4008260 (Supplemental Table S4) seem to be related to the *Yr1* gene, which is effective against the *PstS2* race.

#### 4.2.3 Chromosome 3D

McIntosh et al. (2014) reported *Yr66* to be flanking at approximately 3.0 cM distance to markers IWB47165 and IWB18087/IWB56281 on chromosome 3D, with these markers located at 32,220,594 bp and 3,549,510 bp/3,240,899 bp respectively. The SNP1051462 found in this study was associated with APR located at 519,174,887 bp, indicating a different chromosomal region than *Yr66*. An APR gene *Yr49* and a race‐specific *Yr45* gene were also mapped on chromosome 3D (Li, Chen, Wang, & Jing, 2011; McIntosh et al., 2014). Zegeye et al. (2014) reported a stripe rust seedling resistance SNP *IWA3012_Seedling* mapped near the genomic region of SNP1051462. A SNP in association (FDR ≤ 0.05) with the *Warrior* pathotype is also detected in the same region, hinting at the presence of a potentially race‐specific all‐stage resistance gene. Therefore, it is likely that SNP1051462 is associated with a race‐specific *Yr45* gene.

#### 4.2.4 Chromosome 4A

Two *Yr* genes, *Yr51* and *Yr60*, have previously been reported near the genomic region of SNP2282076 associated with field APR on chromosome 4A. Randhawa et al. (2014) reported *Yr51* on chromosome 4AL as a seedling resistance gene, while Herrera‐Foessel et al. (2015) reported *Yr60* as a moderately effective stripe rust resistance gene, which gives an IT of 4–5 or 5–6 on a 0–9 scale. Since the region was not detected in any of the seedling experiments it is unlikely that SNP2282076 is related to *Yr60*. Another APR QTL was reported in a similar region in an “Avocet/Kariega” population (Prins et al., 2011; Ramburan et al., 2004). The SNP2282076 lies within the proximity of the two APR QTL reported in the the “Avocet/Kariega” population.

#### 4.2.5 Chromosome 4B

Three SNPs, SNP3025627, SNP985169 (Supplemental Table S4) and SNP1200934 (FDR ≤ 0.05) were found to be associated with APR to *Pst* on chromosome 4B. SNP985169 identified on chromosome 4B is in LD with SNP1002449 identified in the seedling experiment with the *Warrior* pathotype, therefore likely tagging the same genomic region. A high‐temperature adult plant (HTAP) resistance gene *Yr62*, which is not expressed at the seedling stage, is reported in this region (Lu et al., 2014). However, this region was also identified in the seedling experiment in association with resistance to the *Warrior* pathotype, therefore likely tagging an all‐stage resistance gene different from *Yr62*. Thus, further genetic analysis is required to confirm their relationship. Naruoka et al. (2015) reported *QYr.wpg‐4B.2 (IWA3994)* as an APR QTL, which overlaps with the SNP3025627 identified in this study, likely tagging the same genomic region. Additionally, an SNP detected in the seedling experiments (FDR ≤ 0.05), in association with resistance to *PstS2*, is in concordance with an earlier reported seedling resistance gene *Yr50* (Liu et al., 2013).

#### 4.2.6 Chromosome 5B

Two APR and one seedling QTL were identified on chromosome 5B. SNP4991320 was found in the region of the previously identified QTL *QYr.tem‐5B.2_Flinor, QYr.caas‐5BL.2_Libellula* and *QYr.inra‐5BL.2_Camp Remy* (Feng et al., 2011; Lu et al., 2009; Mallard et al., 2005). Mallard et al. (2005) and Lu et al. (2009) reported an APR QTL to *Pst* resistance, whereas Feng et al. (2011) reported a temperature sensitive seedling resistance QTL overlapping with SNP4991320. The seedling experiments were not subjected to any temperature change, and the genomic region is not detected in any of the seedling experiments, hinting at an APR QTL. However, further analyses are required to understand the relationship, as SNP4991320 could possibly be related to a temperature sensitive all‐stage resistance QTL.

Similarly, SNP1003602 is identified in the genomic region, where three APR QTL have been previously mapped (Chen et al., 2012; Bansal et al. 2014; Hou et al., 2015). Bansal et al. (2014) reported an APR QTL, while Chen et al. (2012) and Hou et al. (2015) reported a high‐temperature adult‐plant and an all‐stage resistance QTL, respectively. SNP4991320 and SNP1003602 are 131 Mb apart and are not in LD with each other. However, both SNPs overlap many previously reported QTLs for all‐stage, APR and high‐temperature sensitive resistances, which indicates the presence of multiple gene(s)/QTL on chromosome 5B. Bansal et al. (2011) reported a genetic association between *Yr47* and *Lr52* with markers *gwm*234 and *cfb*309 on chromosome 5B, overlapping the region of the SNP1090007 identified in the current study. The *Yr47* gene is reported to confer seedling resistance. Therefore, it is likely that SNP1090007 associated with resistance to the *Warrior* race is linked to *Yr47*.

#### 4.2.7 Chromosome 5D

Stripe rust resistance gene *Yr40/Lr57* is reported on chromosome region 5D (McIntosh et al., 2014). There is no report of *Yr40* being susceptible or resistant to the *PstS2* and/or *Warrior* pathotypes, and we lack the genomic information to compare our findings with the previous reports. However, based on the consensus map (Bulli et al., 2016), SNP2252899 lies significantly outside the genomic region of *Yr40*. The two closest reported QTL to SNP2252899 are *QYrdr.wgp‐5DL (IWA8331)* and *QYr.caas‐5DL (IWA4087)_Zhong 892* (Hou et al., 2015; Liu et al., 2015). *QYr.caas‐5DL (IWA4087)_Zhong 892* was reported as an APR QTL, while *QYrdr.wgp‐5DL (IWA8331)* was reported as a seedling resistance QTL. Since SNP2252899 is a seedling QTL, *QYrdr.wgp‐5DL (IWA8331)* and SNP2252899 seems to be same QTL.

#### 4.2.8 Chromosome 6A

SNP3935577, associated with *PstS2* seedling resistance, was found proximal to the genomic region of *QYr.cim‐6AL_Francolin* (Lan et al., 2014). However, *QYr.cim‐6AL_Francolin* is linked to a minor effect APR QTL, whereas SNP3935577 tags a major effect seedling resistance to *PstS2*. Hence, it is likely that SNP3935577 and *QYr.cim‐6AL_Francolin* are different. Furthermore, two stripe rust resistant genes, *Yr42* and *Yr38*, on the long arm of chromosome 6A have been reported (Marais, Badenhorst, Eksteen, & Pretorius, 2010; Marais, Marais, McCallum, & Pretorius, 2009). *Yr38* is a seedling resistance gene. However, we lack the phenotypic data of the source of *Yr38* to the two *Pst* pathotypes used in present study. Also, genomic information to compare the results identified in our study with the previously reported *Yr*38 gene are lacking. Hence, further analysis is required to establish the relationship between SNP3935577 and the *Yr*38 gene.

#### 4.2.9 Chromosome 6D

The most significant SNP, SNP999763 identified to be associated with the *Warrior* pathotype at the seedling stage was found on chromosome 6D, near the genomic region of previously reported *QYr.ufs‐6D_Cappelle‐Desprez* (Agenbag, Pretorius, Boyd, Bender, & Prins, 2012). Agenbag et al. (2012) reported *QYr.ufs‐6D_Cappelle‐Desprez* as a minor effect APR QTL, whereas SNP999763 exhibited a major effect on seedling resistance. Zegeye et al. (2014) also reported a seedling resistance QTL *IWA4455_Seedling*. Therefore, it is likely that resistance conferred by SNP999763 is due to *IWA4455_Seedling*. In addition, two seedling resistance gene, *Yr20* and *Yr23*, and one APR gene, *YrBai*, have been reported on chromosome 6D (Chen, Jones, & Line, 1995; Ma et al., 2015). Since, no source of *Yr20* and *Yr23* was present in the differential set used in the experiments and without physical genomic positions or a detailed consensus genetic map of different types of markers, it is not possible to establish a relationship of the SNP identified in the current study with any of these previously reported genes, and requires further research.

#### 4.2.10 Chromosome 7A

Two stripe rust resistance associated SNPs i.e. SNP100553682 and SNP3020323, conferring both seedling and adult plant resistance were detected in this genomic region. Two loci, SNP1695008 and SNP1239513, significantly associated (FDR ≤ 0.05) with the *Warrior* pathotype, and associated with field resistance also detected in the same region. All these SNPs were detected in the genomic region of *Yr61, QYr.caas‐7A_Jingshuan16* and *QYr.sun‐7A_CPI133872* (McIntosh et al., 2014; Ren et al., 2012; Zwart et al., 2010). Another high‐temperature seedling resistance gene *Yrxy1* (Zhou et al., 2011) was previously reported on chromosome 7A, but it is unlikely that *Yrxy1* can be the source of resistance, as the seedling experiments were not subjected to any high‐temperature stress. The *Yr61* gene is reported as an all‐stage resistance gene (Zhou et al., 2014), and the QTL detected in the current study reveals that the region confers all‐stage resistance to stripe rust against both *Warrior* and *PstS2*. Therefore, the region is likely related to *Yr61*.

### 4.3 Novel genomic regions identified in this study and their significance

Three genomic regions were tagged, where previously no *Yr* gene or QTL has been reported therefore likely tagging novel genomic regions.

Bulli et al. (2016) reported *QYr.ucw‐1D* at 36.3 Mb on chromosome 1D, whereas the SNP1217081 detected in this study is located at 385.1 Mb. No stripe rust QTL is reported in this region in any previous study, indicating a novel region for APR to stripe rust, as the lines carrying the resistance allele of the SNP were susceptible to both *Pst* races used in the seedling experiments. SNP1217081 was designated as *QYr.1D_APR*.

Two markers, SNP2307897 and SNP5411780, located on chromosome 3A, were found in association with stripe rust resistance to the *Warrior* pathotype at the seedling stage. Gao et al. (2016) reported QTL associated with leaf rust response in the same chromosomal region indicating the potential of this region in chromosome 3A for multiple rust resistance. However, so far no QTL associated with stripe rust is reported in this region of chromosome 3A therefore indicating the region is a potential new source for resistance to stripe rust as well. Both markers, SNP2307897 and SNP5411780 are tagging different genomic regions, as they are not in LD with each other. The closest reported QTL *QYr.cim‐3A_Avocet* (Rosewarne et al., 2012) is 21.7 cM away from SNP2307897 therefore based on the genetic map distances SNPs identified in this study are tagging a novel genomic region. SNP2307897 was designated as *QYr.3A_seedling*.

One marker SNP2245601, designated as *QYr.7D_seedling*, was found in association with seedling resistance against the *PstS2* pathotype on chromosome 7D. Two *Yr* resistance genes *Yr18/Lr34* and *Yr33* are reported on 7D. However, according to consensus maps (Bulli et al., 2016; Maccaferri et al., 2015) SNP2245601 is outside the genomic region of any of these earlier reported genes. The approximate distance between SNP2245601 and *Yr33* at the distal end is 42.5 Mb. Thus, based on the genetic distance SNP2245601 is indicating a novel QTL region.

In light of the scope of the disease the novel genomic regions identified here in this study are of significant importance. Many previous studies have highlighted the importance of landraces and accessions preserved in gene banks for their potential in finding new sources of genes (Naruoka et al., 2015; Muleta et al., 2017). The identification of three novel genomic regions, apart from several already reported significant genomic regions, may be the result of the large genetic diversity present in landraces used in this study. The co‐evolution of landraces along with pathogens over time has enabled them to accumulate diverse resistance loci. This makes them important candidates for study and possible use as donor parents in breeding programs. Although several *Yr* genes have already been reported previously for stripe rust and many of them have been validated in this study, the *Pst* pathogen has the capability to adapt and evolve continuously, breaking down resistance genes. This adaptability has been observed to cause epidemics in regions, which were deemed unfavorable for the disease. Therefore, we need to keep mining new sources of resistance. The impact of rust on the agronomic traits such as yield is still extremely important. Therefore, exploring new sources of resistance is still of paramount importance in rust affected zones to ensure maximum wheat productivity. *PstS2* and *Warrior* are the two most widely spread pathotypes of *Pst*, which are virulent to several important *Yr* genes deployed in the affected regions (Hovmøller et al., 2016; Tadesse et al., 2014). Hence, the information identified here is of high significance, and should be further investigated through the development of functional molecular markers and validation of the QTL by using bi‐parental populations.

## 5 CONCLUSION

The results of the current study emphasize the prospects of taking advantage of high genetic diversity in bread wheat landraces mainly due to historic recombination. The landraces preserved at the ICARDA gene bank possess a wide range of phenotypic diversity for both seedling stage resistance and APR to wheat stripe rust. Accessions with a higher resistance response across seedling and adult‐plant stages can be vital genetic material in breeding programs against stripe rust, and the molecular markers linked to resistance QTL could serve as reliable breeding tools for future wheat breeding programs addressing stripe rust. This result provided the baseline for the next phase of the study, which is to utilize the marker sequences and convert them into functional markers, which then will be utilized to validate the QTL by using bi‐parental populations, Recombinant inbred lines (RILs) or near‐isogenic lines (NILs).

## CONFLICT OF INTEREST DISCLOSURE

The authors declare that there is no conflict of interest.

## Supporting information

Supplemental Figure. S1. The LD decay plots of wheat genome A, B, and D.

Supplemental Figure. S2. The 600 × 600 kinship matrix based on simple matching genetic similarities (IBS, identity by state).

Supplemental Table S1. List of bread wheat landraces collected from ICARDA gene bank used in the study including their country of origin.

Supplemental Table S2. Virulence/Avirulence formula for the *PstS2* and *Warrior* Pathotypes of *Pst*.

Supplemental Table S3. Differential set used in the study for race typing.

Supplemental Table S4. List of SNPs associated (marker‐wise p ≤ .001) to both seedling and field‐based resistance to stripe rust.

Supplemental Table S5. Adult‐plant and seedling phenotypic data of 600 bread wheat landraces used in GWAS analysis.

Supplemental Table. S6. Genotypic data of 600 bread wheat landraces.

Supplemental File. S1. Relationship of significant genomic regions (FDR (q) ≤ 0.05) associated with seedling and field resistance with previously identified *Yr* genes or QTL.

## References

[tpg220066-bib-0001] Agenbag, G. M. , Pretorius, Z. A. , Boyd, L. A. , Bender, C. M. , & Prins, R. (2012). Identification of adult plant resistance to stripe rust in the wheat cultivar Cappelle‐Desprez. Theoretical and Applied Genetics, 125(1), 109–120. 10.1007/s00122-012-1819-5 22350093

[tpg220066-bib-0002] Ain, Q. U. , Rasheed, A. , Anwar, A. , Mahmood, T. , Imtiaz, M. , He, Z. , … Quraishi, U. M. (2015). Genome‐wide association for grain yield under rainfed conditions in historical wheat cultivars from Pakistan. Frontiers in Plant Science, 6, 743. 10.3389/fpls.2015.00743 26442056 PMC4585131

[tpg220066-bib-0003] Akçura, M. (2011). The relationships of some traits in Turkish winter bread wheat landraces. Turkish Journal of Agriculture and Forestry, 35(2), 115–125.

[tpg220066-bib-0004] Akcura, M. , Kadir, A. K. A. N. , & Hocaoglu, O. (2017). Biplot analysis of leaf rust resistance in pure lines selected from eastern Anatolian bread wheat landraces of turkey. Turkish Journal of Field Crops, 22(2), 227–234.

[tpg220066-bib-0005] Ali, S. , Gladieux, P. , Leconte, M. , Gautier, A. , Justesen, A. F. , Hovmøller, M. S. , … de Vallavieille‐Pope, C. (2014). Origin, migration routes and worldwide population genetic structure of the wheat yellow rust pathogen *Puccinia striiformis* f. sp. *tritici* . PLoS Pathogen, 10(1), e1003903. 24465211 10.1371/journal.ppat.1003903 PMC3900651

[tpg220066-bib-0006] Ali, S. , Rodriguez‐Algaba, J. , Thach, T. , Sørensen, C. K. , Hansen, J. G. , Lassen, P. , … Hovmøller, M. S. (2017). Yellow rust epidemics worldwide were caused by pathogen races from divergent genetic lineages. Frontiers in Plant Science, 8, 1057. 10.3389/fpls.2017.01057 28676811 PMC5477562

[tpg220066-bib-0007] Appels, R. , Eversole, K. , Stein, N. , Feuillet, C. , Keller, B. , Rogers, J. , … & Ronen, G. (2018). Shifting the limits in wheat research and breeding using a fully annotated reference genome. Science, 361(6403). 10.1126/science.aar7191 30115783

[tpg220066-bib-0008] Bansal, U. K. , Forrest, K. L. , Hayden, M. J. , Miah, H. , Singh, D. , & Bariana, H. S. (2011). Characterisation of a new stripe rust resistance gene Yr47 and its genetic association with the leaf rust resistance gene Lr52. Theoretical and Applied Genetics, 122(8), 1461–1466. 10.1007/s00122-011-1545-4 21344185

[tpg220066-bib-0016] Bansal, U. K. , Kazi, A. G. , Singh, B. , Hare, R. A. , & Bariana, H. S . (2014). Mapping of durable stripe rust resistance in a durum wheat cultivar Wollaroi. Molecular Breeding, 33(1), 51–59.

[tpg220066-bib-0009] Benjamini, Y. , & Hochberg, Y. (1995). Controlling the false discovery rate: A practical and powerful approach to multiple testing. Journal of the Royal Statistical Society: Series B (Methodological), 57(1), 289–300.

[tpg220066-bib-0010] Benson, J. , Brown‐Guedira, G. , Paul Murphy, J. , & Sneller, C. (2012). Population structure, linkage disequilibrium, and genetic diversity in soft winter wheat enriched for fusarium head blight resistance. The Plant Genome, 5(2), 71–80. 10.3835/plantgenome2011.11.0027

[tpg220066-bib-0011] Bentley, A. R. , Scutari, M. , Gosman, N. , Faure, S. , Bedford, F. , Howell, P. , … Horsnell, R. (2014). Applying association mapping and genomic selection to the dissection of key traits in elite European wheat. Theoretical and Applied Genetics, 127(12), 2619–2633. 10.1007/s00122-014-2403-y 25273129

[tpg220066-bib-0012] Bird, M. (1999). Ever nearer the past. Time Magazine, 154, 64–71.10620923

[tpg220066-bib-0013] Bonman, J. M. , Bockelman, H. E. , Goates, B. J. , Obert, D. E. , McGuire, P. E. , Qualset, C. O. , & Hijmans, R. J. (2006). Geographic distribution of common and dwarf bunt resistance in landraces of Triticum aestivum subsp. aestivum. Crop Science, 46(4), 1622–1629. 10.2135/cropsci2005.12-0463

[tpg220066-bib-0014] Boukhatem, N. , Baret, P. V. , Mingeot, D. , & Jacquemin, J. M. (2002). Quantitative trait loci for resistance against yellow rust in two wheat‐derived recombinant inbred line populations. Theoretical and Applied Genetics, 104(1), 111–118. 10.1007/s001220200013 12579435

[tpg220066-bib-0015] Bradbury, P. J. , Zhang, Z. , Kroon, D. E. , Casstevens, T. M. , Ramdoss, Y. , & Buckler, E. S. (2007). TASSEL: Software for association mapping of complex traits in diverse samples. Bioinformatics, 23(19), 2633–2635. 10.1093/bioinformatics/btm308 17586829

[tpg220066-bib-0017] Bulli, P. , Zhang, J. , Chao, S. , Chen, X. , & Pumphrey, M. (2016). Genetic architecture of resistance to stripe rust in a global winter wheat germplasm collection. G3: Genes, Genomes, Genetics, 6(8), 2237–2253. 10.1534/g3.116.028407 27226168 PMC4978880

[tpg220066-bib-0018] Burdon, J. J. , Barrett, L. G. , Rebetzke, G. , & Thrall, P. H. (2014). Guiding deployment of resistance in cereals using evolutionary principles. Evolutionary Applications, 7(6), 609–624. 10.1111/eva.12175 25067946 PMC4105914

[tpg220066-bib-0019] Chao, S. , Zhang, W. , Dubcovsky, J. , & Sorrells, M. (2007). Evaluation of genetic diversity and genome‐wide linkage disequilibrium among US wheat (Triticum aestivum L.) germplasm representing different market classes. Crop Science, 47(3), 1018–1030. 10.2135/cropsci2006.06.0434

[tpg220066-bib-0020] Chen, G. , Long, L. , Yao, F. , Yu, C. , Ye, X. , Cheng, Y. , … Chen, G. (2019). Genome‐wide association study for adult‐plant resistance to stripe rust in chinese wheat landraces (Triticum aestivum L.) from the yellow and huai river valleys. Frontiers in Plant Science, 10, 596. 31156668 10.3389/fpls.2019.00596 PMC6532019

[tpg220066-bib-0021] Chen, J. , Chu, C. , Souza, E. J. , Guttieri, M. J. , Chen, X. , Xu, S. , & Zemetra, R. (2012). Genome‐wide identification of QTL conferring high‐temperature adult‐plant (HTAP) resistance to stripe rust (*Puccinia striiformis* f. sp. *tritici*) in wheat. Molecular Breeding, 29(3), 791–800.

[tpg220066-bib-0022] Chen, X. M. , Jones, S. S. , & Line, R. F. (1995). Chromosomal location of genes for stripe rust resistance in spring wheat cultivars Compair, Fielder, Lee, and Lemhi and interactions of aneuploid wheats with races of *Puccinia striiformis* . Phytopathology, 85(3), 375–381. 10.1094/Phyto-85-375

[tpg220066-bib-0023] Chen, X. (2013). High‐temperature adult‐plant resistance, key for sustainable control of stripe rust. American Journal of Plant Sciences, 4(03), 608. 10.4236/ajps.2013.43080

[tpg220066-bib-0024] Chen, X. M. (2005). Epidemiology and control of stripe rust [*Puccinia striiformis* f. sp. *tritici*] on wheat. Canadian Journal of Plant Pathology, 27(3), 314–337. 10.1080/07060660509507230

[tpg220066-bib-0094] Core Team R. (2013). R: A language and environment for statistical computing. Vienna, Austria: R Core Team.

[tpg220066-bib-0025] Crossa, J. , Burgueno, J. , Dreisigacker, S. , Vargas, M. , Herrera‐Foessel, S. A. , Lillemo, M. , … Reynolds, M. (2007). Association analysis of historical bread wheat germplasm using additive genetic covariance of relatives and population structure. Genetics, 177(3), 1889–1913. 10.1534/genetics.107.078659 17947425 PMC2147943

[tpg220066-bib-0026] Dinesh, A. , Patil, A. , Zaidi, P. H. , Kuchanur, P. H. , Vinayan, M. T. , & Seetharam, K. (2016). Genetic diversity, linkage disequilibrium and population structure among CIMMYT maize inbred lines, selected for heat tolerance study. Maydica, 61(M29).

[tpg220066-bib-0027] Doyle, J. J. , & Doyle, J. L. (1990). Isolation of plant DNA from fresh tissue. Focus, 12(13), 39–40.

[tpg220066-bib-0028] Ellis, J. G. , Lagudah, E. S. , Spielmeyer, W. , & Dodds, P. N. (2014). The past, present and future of breeding rust resistant wheat. Frontiers in Plant Science, 5, 641. 10.3389/fpls.2014.00641 25505474 PMC4241819

[tpg220066-bib-0029] Endresen, F. , Terje, D. , Street, K. , Mackay, M. , Bari, A. , & De Pauw, E. (2011). Predictive association between biotic stress traits and eco‐geographic data for wheat and barley landraces. Crop Science, 51(5), 2036–2055. 10.2135/cropsci2010.12.0717

[tpg220066-bib-0030] Evanno, G. , Regnaut, S. , & Goudet, J. (2005). Detecting the number of clusters of individuals using the software STRUCTURE: a simulation study. Molecular Ecology, 14(8), 2611–2620. 15969739 10.1111/j.1365-294X.2005.02553.x

[tpg220066-bib-0031] Feng, J. , Zuo, L. L. , Zhang, Z. Y. , Lin, R. M. , Cao, Y. Y. , & Xu, S. C. (2011). Quantitative trait loci for temperature‐sensitive resistance to *Puccinia striiformis* f. sp. *tritici* in wheat cultivar Flinor. Euphytica, 178(3), 321–329. 10.1007/s10681-010-0291-z

[tpg220066-bib-0032] Gao, L. , Turner, M. K. , Chao, S. , Kolmer, J. , & Anderson, J. A. (2016). Genome‐wide association study of seedling and adult plant leaf rust resistance in elite spring wheat breeding lines. PLoS One, 11(2), e0148671. 10.1371/journal.pone.0148671 26849364 PMC4744023

[tpg220066-bib-0033] Gurung, S. , Mamidi, S. , Bonman, J. M. , Xiong, M. , Brown‐Guedira, G. , & Adhikari, T. B. (2014). Genome‐wide association study reveals novel quantitative trait loci associated with resistance to multiple leaf spot diseases of spring wheat. PLoS One, 9(9), e108179. 10.1371/journal.pone.0108179 25268502 PMC4182470

[tpg220066-bib-0034] Herrera‐Foessel, S. A. , Singh, R. P. , Lan, C. X. , Huerta‐Espino, J. , Calvo‐Salazar, V. , Bansal, U. K. , & Lagudah, E. S. (2015). Yr60, a gene conferring moderate resistance to stripe rust in wheat. Plant Disease, 99(4), 508–511. 10.1094/PDIS-08-14-0796-RE 30699549

[tpg220066-bib-0035] Hoisington, D. A. , Khairallah, M. M. , & González de León, D. (1994). *Laboratory protocols* (No. CIM 0410‐R 1994 En. CIMMYT). Centro Internacional de Mejoramiento de Maiz y Trigo (CIMMYT), Mexico DF.

[tpg220066-bib-0036] Hou, L. , Chen, X. , Wang, M. , See, D. R. , Chao, S. , Bulli, P. , & Jing, J. (2015). Mapping a large number of QTL for durable resistance to stripe rust in winter wheat Druchamp using SSR and SNP markers. PLoS One, 10(5), e0126794. 10.1371/journal.pone.0126794 25970329 PMC4430513

[tpg220066-bib-0037] Hovmøller, M. S. , Sørensen, C. K. , Walter, S. , & Justesen, A. F. (2011). Diversity of *Puccinia striiformis* on cereals and grasses. Annual Review of Phytopathology, 49, 197–217. 10.1146/annurev-phyto-072910-095230 21599494

[tpg220066-bib-0038] Hovmøller, M. S. , Walter, S. , Bayles, R. A. , Hubbard, A. , Flath, K. , Sommerfeldt, N. , & Hansen, J. G. (2016). Replacement of the European wheat yellow rust population by new races from the centre of diversity in the near‐Himalayan region. Plant Pathology, 65(3), 402–411. 10.1111/ppa.12433

[tpg220066-bib-0039] Huang, X. , Sang, T. , Zhao, Q. , Feng, Q. , Zhao, Y. , Li, C. , & Fan, D. (2010). Genome‐wide association studies of 14 agronomic traits in rice landraces. Nature Genetics, 42(11), 961. 10.1038/ng.695 20972439

[tpg220066-bib-0040] Hulbert, S. , & Pumphrey, M. (2014). A time for more booms and fewer busts? Unraveling cereal–rust interactions. Molecular Plant‐Microbe Interactions, 27(3), 207–214. 10.1094/MPMI-09-13-0295-FI 24499028

[tpg220066-bib-0041] Jighly, A. , Oyiga, B. C. , Makdis, F. , Nazari, K. , Youssef, O. , Tadesse, W. , & Ogbonnaya, F. C. (2015). Genome‐wide DArT and SNP scan for QTL associated with resistance to stripe rust (*Puccinia striiformis* f. sp. *tritici*) in elite ICARDA wheat (*Triticumaestivum* L.) germplasm. Theoretical and Applied Genetics, 128(7), 1277–1295. 10.1007/s00122-015-2504-2 25851000

[tpg220066-bib-0042] Juliana, P. , Singh, R. P. , Singh, P. K. , Poland, J. A. , Bergstrom, G. C. , Huerta‐Espino, J. , … Sorrells, M. E. (2018). Genome‐wide association mapping for resistance to leaf rust, stripe rust and tan spot in wheat reveals potential candidate genes. Theoretical and Applied Genetics, 131(7), 1405–1422. 10.1007/s00122-018-3086-6 29589041 PMC6004277

[tpg220066-bib-0043] Karagöz, A. (2014). Wheat landraces of Turkey. Emirates Journal of Food and Agriculture, 26(2), 149.

[tpg220066-bib-0044] Kertho, A. , Mamidi, S. , Bonman, J. M. , McClean, P. E. , & Acevedo, M. (2015). Genome‐wide association mapping for resistance to leaf and stripe rust in winter‐habit hexaploid wheat landraces. PLoS One, 10(6), e0129580. 10.1371/journal.pone.0129580 26076040 PMC4468153

[tpg220066-bib-0045] Lan, C. , Rosewarne, G. M. , Singh, R. P. , Herrera‐Foessel, S. A. , Huerta‐Espino, J. , Basnet, B. R. , & Yang, E. (2014). QTL characterization of resistance to leaf rust and stripe rust in the spring wheat line Francolin 1. Molecular Breeding, 34(3), 789–803.

[tpg220066-bib-0046] Li, Q. , Chen, X. M. , Wang, M. N. , & Jing, J. X. (2011). Yr45, a new wheat gene for stripe rust resistance on the long arm of chromosome 3D. Theoretical and Applied Genetics, 122(1), 189–197. 10.1007/s00122-010-1435-1 20838759

[tpg220066-bib-0047] Lillemo, M. , Asalf, B. , Singh, R. P. , Huerta‐Espino, J. , Chen, X. M. , He, Z. H. , & Bjørnstad, Å (2008). The adult plant rust resistance loci Lr34/Yr18 and Lr46/Yr29 are important determinants of partial resistance to powdery mildew in bread wheat line Saar. Theoretical and Applied Genetics, 116(8), 1155–1166. 10.1007/s00122-008-0743-1 18347772

[tpg220066-bib-0048] Lipka, A. E. , Tian, F. , Wang, Q. , Peiffer, J. , Li, M. , Bradbury, P. J. , & Zhang, Z. (2012). GAPIT: Genome association and prediction integrated tool. Bioinformatics, 28(18), 2397–2399. 10.1093/bioinformatics/bts444 22796960

[tpg220066-bib-0049] Liu, J. , Chang, Z. , Zhang, X. , Yang, Z. , Li, X. , Jia, J. , & Wang, J. (2013). Putative Thinopyrum intermedium‐derived stripe rust resistance gene Yr50 maps on wheat chromosome arm 4BL. Theoretical and Applied Genetics, 126(1), 265–274. 10.1007/s00122-012-1979-3 23052018

[tpg220066-bib-0050] Liu, J. , He, Z. , Wu, L. , Bai, B. , Wen, W. , Xie, C. , & Xia, X. (2015). Genome‐wide linkage mapping of QTL for adult‐plant resistance to stripe rust in a Chinese wheat population Linmai 2 × Zhong 892. PloS one, 10(12), e0145462. 10.1371/journal.pone.0145462 26714310 PMC4694644

[tpg220066-bib-0051] Liu, W. , Maccaferri, M. , Rynearson, S. , Letta, T. , Zegeye, H. , Tuberosa, R. , & Pumphrey, M. (2017). Novel sources of stripe rust resistance identified by genome‐wide association mapping in Ethiopian durum wheat (*Triticum turgidum* ssp. *durum*). Frontiers in Plant Science, 8, 774. 10.3389/fpls.2017.00774 28553306 PMC5427679

[tpg220066-bib-0052] Lu, Y. , Lan, C. , Liang, S. , Zhou, X. , Liu, D. , Zhou, G. , & He, Z. (2009). QTL mapping for adult‐plant resistance to stripe rust in Italian common wheat cultivars Libellula and Strampelli. Theoretical and Applied Genetics, 119(8), 1349–1359. 10.1007/s00122-009-1139-6 19756474

[tpg220066-bib-0053] Lu, Y. , Wang, M. , Chen, X. , See, D. , Chao, S. , & Jing, J. (2014). Mapping of Yr62 and a small‐effect QTL for high‐temperature adult‐plant resistance to stripe rust in spring wheat PI 192252. Theoretical and Applied Genetics, 127(6), 1449–1459. 10.1007/s00122-014-2312-0 24781075

[tpg220066-bib-0054] Luu, K. , Bazin, E. , & Blum, M. G. (2017). pcadapt: An R package to perform genome scans for selection based on principal component analysis. Molecular Ecology Resources, 17(1), 67–77. 10.1111/1755-0998.12592 27601374

[tpg220066-bib-0055] Ma, D. , Li, Q. , Tang, M. , Chao, K. , Li, J. , Wang, B. , & Jing, J. (2015). Mapping of gene conferring adult‐plant resistance to stripe rust in Chinese wheat landrace Baidatou. Molecular Breeding, 35(8), 157. 10.1007/s11032-015-0244-2

[tpg220066-bib-0056] Maccaferri, M. , Zhang, J. , Bulli, P. , Abate, Z. , Chao, S. , Cantu, D. , & Dubcovsky, J. (2015). A genome‐wide association study of resistance to stripe rust (*Puccinia striiformis* f. sp. *tritici*) in a worldwide collection of hexaploid spring wheat (*Triticum aestivum* L.). G3: Genes, Genomes, Genetics, 5(3), 449–465. 10.1534/g3.114.014563 25609748 PMC4349098

[tpg220066-bib-0057] Mallard, S. , Gaudet, D. , Aldeia, A. , Abelard, C. , Besnard, A. L. , Sourdille, P. , & Dedryver, F. (2005). Genetic analysis of durable resistance to yellow rust in bread wheat. Theoretical and Applied Genetics, 110(8), 1401–1409. 10.1007/s00122-005-1954-3 15841362

[tpg220066-bib-0058] Manickavelu, A. , Joukhadar, R. , Jighly, A. , Lan, C. , Huerta‐Espino, J. , Stanikzai, A. S. , & Ban, T. (2016). Genome wide association mapping of stripe rust resistance in Afghan wheat landraces. Plant Science, 252, 222–229. 10.1016/j.plantsci.2016.07.018 27717458

[tpg220066-bib-0059] Marais, G. F. , Badenhorst, P. E. , Eksteen, A. , & Pretorius, Z. A. (2010). Reduction of Aegilops sharonensis chromatin associated with resistance genes Lr56 and Yr38 in wheat. Euphytica, 171(1), 15–22. 10.1007/s10681-009-9973-9

[tpg220066-bib-0060] Marais, F. , Marais, A. , McCallum, B. , & Pretorius, Z. (2009). Transfer of leaf rust and stripe rust resistance genes Lr62 and Yr42 from Aegilops neglecta Req. ex Bertol. to common wheat. Crop Science, 49(3), 871–879. 10.2135/cropsci2008.06.0317

[tpg220066-bib-0061] Mather, K. A. , Caicedo, A. L. , Polato, N. R. , Olsen, K. M. , McCouch, S. , & Purugganan, M. D. (2007). The extent of linkage disequilibrium in rice (*Oryza sativa* L.). Genetics, 177(4), 2223–2232. 10.1534/genetics.107.079616 17947413 PMC2219496

[tpg220066-bib-0062] McIntosh, R. A. , Dubcovsky, J. , Rogers, J. W. , Morris, C. F. , Appels, R. , & Xia, X. (2014). Catalogue of gene symbols for wheat: 2013–14 Supplement. Annual Wheat Newsletter, 58.

[tpg220066-bib-0063] McNeal, F. H. , Konzak, C. F. , Smith, E. P. , Tate, W. S. , & Russell, T. S. (1971). *A uniform system for recording and processing cereal research data*. No. REP‐10904. CIMMYT, Mexico DF.

[tpg220066-bib-0064] Melichar, J. P. E. , Berry, S. , Newell, C. , MacCormack, R. , & Boyd, L. A. (2008). QTL identification and microphenotype characterisation of the developmentally regulated yellow rust resistance in the UK wheat cultivar Guardian. Theoretical and Applied Genetics, 117(3), 391. 10.1007/s00122-008-0783-6 18481042

[tpg220066-bib-0065] Mert, Z. , Nazari, K. , Karagöz, E. , Akan, K. , Öztürk, İ. , & Tülek, A. (2016). First incursion of the warrior race of wheat stripe rust (*Puccinia striiformis* f. sp. *tritici*) to Turkey in 2014. Plant Diseases, 100(2), 528. 10.1094/PDIS-07-15-0827-PDN

[tpg220066-bib-0066] Morgounov, A. , Keser, M. , Kan, M. , Küçükçongar, M. , Özdemir, F. , Gummadov, N. , & Qualset, C. O. (2016). Wheat landraces currently grown in Turkey: Distribution, diversity, and use. Crop Science, 56(6), 3112–3124. 10.2135/cropsci2016.03.0192

[tpg220066-bib-0067] Muleta, K. T. , Bulli, P. , Rynearson, S. , Chen, X. , & Pumphrey, M. (2017). Loci associated with resistance to stripe rust (*Puccinia striiformis* f. sp. *tritici*) in a core collection of spring wheat (*Triticum aestivum*). PloS one, 12(6), e0179087. 10.1371/journal.pone.0179087 28591221 PMC5462451

[tpg220066-bib-0068] Mumtaz, S. , Khan, I. A. , Ali, S. , Zeb, B. , Iqbal, A. , Shah, Z. , & Swati, Z. A. (2009). Development of RAPD based markers for wheat rust resistance gene cluster (Lr37‐Sr38‐Yr17) derived from *Triticum ventricosum* L. African Journal of Biotechnology, 8(7).

[tpg220066-bib-0069] Naruoka, Y. , Garland‐Campbell, K. A. , & Carter, A. H. (2015). Genome‐wide association mapping for stripe rust (*Puccinia striiformis* F. sp. *tritici*) in US Pacific Northwest winter wheat (*Triticum aestivum* L.). Theoretical and Applied Genetics, 128(6), 1083–1101. 10.1007/s00122-015-2492-2 25754424

[tpg220066-bib-0070] Ozkuru, E. , Ates, D. , Nemli, S. , Erdogmus, S. , Karaca, N. , Yilmaz, H. , & Otles, S. (2019). Association mapping of loci linked to copper, phosphorus, and potassium concentrations in the seeds of *C. arietinum* and *C. reticulatum* . Genomics, 111(6), 1873–1881. 10.1016/j.ygeno.2018.12.010 30594584

[tpg220066-bib-0071] Patterson, H. D. , & Thompson, R. (1971). Recovery of inter‐block information when block sizes are unequal. Biometrika, 58(3), 545–554. 10.1093/biomet/58.3.545

[tpg220066-bib-0072] Peterson, R. F. , Campbell, A. B. , & Hannah, A. E. (1948). A diagrammatic scale for estimating rust intensity on leaves and stems of cereals. Canadian Journal of Research, 26(5), 496–500. 10.1139/cjr48c-033

[tpg220066-bib-0073] Prins, R. , Pretorius, Z. A. , Bender, C. M. , & Lehmensiek, A. (2011). QTL mapping of stripe, leaf and stem rust resistance genes in a Kariega × Avocet S doubled haploid wheat population. Molecular Breeding, 27(2), 259–270.

[tpg220066-bib-0074] Pritchard, J. K. , Stephens, M. , & Donnelly, P. (2000). Inference of population structure using multilocus genotype data. Genetics, 155(2), 945–959. 10835412 10.1093/genetics/155.2.945 PMC1461096

[tpg220066-bib-0075] Rahmatov, M. (2016). Genetic characterisation of novel resistance alleles to stem rust and stripe rust in wheat‐alien introgression lines. Doctoral dissertation. Department of Plant Breeding, Swedish University of Agricultural Sciences.

[tpg220066-bib-0076] Ramburan, V. P. , Pretorius, Z. A. , Louw, J. H. , Boyd, L. A. , Smith, P. H. , Boshoff, W. H. P. , & Prins, R. (2004). A genetic analysis of adult plant resistance to stripe rust in the wheat cultivar Kariega. Theoretical and Applied Genetics, 108(7), 1426–1433. 10.1007/s00122-003-1567-7 14963651

[tpg220066-bib-0077] Randhawa, M. , Bansal, U. , Valárik, M. , Klocová, B. , Doležel, J. , & Bariana, H. (2014). Molecular mapping of stripe rust resistance gene Yr51 in chromosome 4AL of wheat. Theoretical and Applied Genetics, 127(2), 317–324. 10.1007/s00122-013-2220-8 24185819

[tpg220066-bib-0078] Ren, Y. , Li, Z. , He, Z. , Wu, L. , Bai, B. , Lan, C. … & Xia, X. (2012). QTL mapping of adult‐plant resistances to stripe rust and leaf rust in Chinese wheat cultivar Bainong 64. Theoretical and Applied Genetics, 125(6), 1253–1262. 10.1007/s00122-012-1910-y 22806327

[tpg220066-bib-0079] Roelfs, A. P. (1992). *Rust diseases of wheat: Concepts and methods of disease management*. CIMMYT, Mexico DF.

[tpg220066-bib-0080] Rosewarne, G. M. , Singh, R. P. , Huerta‐Espino, J. , Herrera‐Foessel, S. A. , Forrest, K. L. , Hayden, M. J. , & Rebetzke, G. J. (2012). Analysis of leaf and stripe rust severities reveals pathotype changes and multiple minor QTLs associated with resistance in an Avocet × Pastor wheat population. Theoretical and Applied Genetics, 124(7), 1283–1294. 22274764 10.1007/s00122-012-1786-x

[tpg220066-bib-0081] Rosewarne, G. M. , Singh, R. P. , Huerta‐Espino, J. , William, H. M. , Bouchet, S. , Cloutier, S. , & Lagudah, E. S. (2006). Leaf tip necrosis, molecular markers and β1‐proteasome subunits associated with the slow rusting resistance genes Lr46/Yr29. Theoretical and Applied Genetics, 112(3), 500–508. 10.1007/s00122-005-0153-6 16331478

[tpg220066-bib-0082] Saari, E. E. , & Wilcoxson, R. D. (1974). Plant disease situation of high‐yielding dwarf wheats in Asia and Africa. Annual Review of Phytopathology, 12(1), 49–68. 10.1146/annurev.py.12.090174.000405

[tpg220066-bib-0083] Sajjad, M. , Khan, S. H. , Ahmad, M. Q. , Rasheed, A. , Mujeeb‐Kazi, A. , & Khan, I. A. (2014). Association mapping identifies QTLs on wheat chromosome 3A for yield related traits. Cereal Research Communications, 42(2), 177–188. 10.1556/CRC.2013.0061

[tpg220066-bib-0084] Sansaloni, C. , Petroli, C. , Jaccoud, D. , Carling, J. , Detering, F. , Grattapaglia, D. , & Kilian, A. (2011). Diversity Arrays Technology (DArT) and next‐generation sequencing combined: Genome‐wide, high throughput, highly informative genotyping for molecular breeding of Eucalyptus. BMC Proceedings, 5(7), 54.

[tpg220066-bib-0085] Sehgal, D. , Dreisigacker, S. , Belen, S. , Küçüközdemir, Ü. , Mert, Z. , Özer, E. , & Morgounov, A. (2016). Mining centuries old in situ conserved Turkish wheat landraces for grain yield and stripe rust resistance genes. Frontiers in Genetics, 7, 201. 10.3389/fgene.2016.00201 27917192 PMC5114521

[tpg220066-bib-0086] Singh, R. P. (2001). Slow rusting genes based resistance to leaf and yellow rusts in wheat: Genetics and breeding at CIMMYT. *Proceedings of the 10th Assembly of the Wheat Breeding Society of Australia*. Mildura, Australia, 16–21 September 2001, 103–108.

[tpg220066-bib-0087] Singh, R. P. , Huerta‐Espino, J. U. L. I. O. , & William, H. M. (2005). Genetics and breeding for durable resistance to leaf and stripe rusts in wheat. Turkish Journal of Agriculture and Forestry, 29(2), 121–127.

[tpg220066-bib-0088] Singh, S. , Ledesma‐Ramírez, L. , Solís‐Moya, E. , Iturriaga, G. , Sehgal, D. , Reyes‐Valdes, M. H. , … Aguirre‐Mancilla, C. L. (2019). GWAS to identify genetic loci for resistance to yellow rust in wheat pre‐breeding lines derived from diverse exotic crosses. Frontiers in Plant Science, 10, 1390. 31781137 10.3389/fpls.2019.01390 PMC6831551

[tpg220066-bib-0089] Singh, R. P. , Huerta‐Espino, J. , & Rajaram, S. (2000). Achieving near‐immunity to leaf and stripe rusts in wheat by combining slow rusting resistance genes. Acta phytopathologica et entomologica hungarica, 35(1/4), 133–139.

[tpg220066-bib-0090] Solh, M. , Nazari, K. , Tadesse, W. , & Wellings, C. R. (2012). The growing threat of stripe rust worldwide. Borlaug Global Rust Initiative (BGRI) Conference . Beijing, China, 1–4.

[tpg220066-bib-0091] Sørensen, C. K. , Hovmøller, M. S. , Leconte, M. , Dedryver, F. , & de Vallavieille‐Pope, C. (2014). New races of *Puccinia striiformis* found in Europe reveal race specificity of long‐term effective adult plant resistance in wheat. Phytopathology, 104(10), 1042–1051. 10.1094/PHYTO-12-13-0337-R 24624957

[tpg220066-bib-0092] Steele, K. A. , Humphreys, E. , Wellings, C. R. , & Dickinson, M. J. (2001). Support for a stepwise mutation model for pathogen evolution in Australasian *Puccinia striiformis* f. sp. *tritici* by use of molecular markers. Plant Pathology, 50(2), 174–180. 10.1046/j.1365-3059.2001.00558.x

[tpg220066-bib-0093] Tadesse, W. , Ogbonnaya, F. C. , Jighly, A. , Nazari, K. , Rajaram, S. , & Baum, M. (2014). Association mapping of resistance to yellow rust in winter wheat cultivars and elite genotypes. Crop Science, 54(2), 607–616. 10.2135/cropsci2013.05.0289

[tpg220066-bib-0095] Vos, P. G. , Paulo, M. J. , Voorrips, R. E. , Visser, R. G. , van Eck, H. J. , & van Eeuwijk, F. A. (2017). Evaluation of LD decay and various LD‐decay estimators in simulated and SNP‐array data of tetraploid potato. Theoretical and Applied Genetics, 130(1), 123–135. 10.1007/s00122-016-2798-8 27699464 PMC5214954

[tpg220066-bib-0096] William, M. , Singh, R. P. , Huerta‐Espino, J. , Islas, S. O. , & Hoisington, D. (2003). Molecular marker mapping of leaf rust resistance gene Lr46 and its association with stripe rust resistance gene Yr29 in wheat. Phytopathology, 93(2), 153–159. 10.1094/PHYTO.2003.93.2.153 18943129

[tpg220066-bib-0097] Yao, F. , Zhang, X. , Ye, X. , Li, J. , Long, L. , Yu, C. , & Jiang, Q. (2019). Characterization of molecular diversity and genome‐wide association study of stripe rust resistance at the adult plant stage in Northern Chinese wheat landraces. BMC Genetics, 20(1), 38. 10.1186/s12863-019-0736-x 30914040 PMC6434810

[tpg220066-bib-0098] Yu, H. , Deng, Z. , Xiang, C. , & Tian, J. (2014). Analysis of diversity and linkage disequilibrium mapping of agronomic traits on B‐genome of wheat. Journal of Genomics, 2, 20. 10.7150/jgen.4089 25057321 PMC4105426

[tpg220066-bib-0099] Zegeye, H. , Rasheed, A. , Makdis, F. , Badebo, A. , & Ogbonnaya, F. C. (2014). Genome‐wide association mapping for seedling and adult plant resistance to stripe rust in synthetic hexaploid wheat. PLoS One, 9(8), e105593. 10.1371/journal.pone.0105593 25153126 PMC4143293

[tpg220066-bib-0100] Zeven, A. C. (1998). Landraces: A review of definitions and classifications. Euphytica, 104(2), 127–139. 10.1023/A:1018683119237

[tpg220066-bib-0101] Zhou, X. L. , Han, D. J. , Chen, X. M. , Gou, H. L. , Guo, S. J. , Rong, L. , & Kang, Z. S. (2014). Characterization and molecular mapping of stripe rust resistance gene Yr61 in winter wheat cultivar Pindong 34. Theoretical and Applied Genetics, 127(11), 2349–2358. 10.1007/s00122-014-2381-0 25163935

[tpg220066-bib-0102] Zhou, X. L. , Wang, W. L. , Wang, L. L. , Hou, D. Y. , Jing, J. X. , Wang, Y. , & Ma, D. F. (2011). Genetics and molecular mapping of genes for high‐temperature resistance to stripe rust in wheat cultivar Xiaoyan 54. Theoretical and Applied Genetics, 123(3), 431–438. 10.1007/s00122-011-1595-7 21516354

[tpg220066-bib-0103] Zwart, R. S. , Thompson, J. P. , Milgate, A. W. , Bansal, U. K. , Williamson, P. M. , Raman, H. , & Bariana, H. S. (2010). QTL mapping of multiple foliar disease and root‐lesion nematode resistances in wheat. Molecular Breeding, 26(1), 107–124. 10.1007/s11032-009-9381-9

