# Supplementary material for: Genome‐wide association study of resistance to PstS2 and Warrior races of Puccinia striiformis f. sp. tritici (stripe rust) in bread wheat landraces
Source: Plant Genome. 2020 Dec 7;14(1):e20066. doi: 10.1002/tpg2.20066 (PMC12807014; doi:10.1002/tpg2.20066)
Supplement: Supplementary file 1 [file TPG2-14-e20066-s006.docx]

GENOME WIDE ASSOCIATION STUDY OF RESISTANCE TO PSTS2 AND WARRIOR RACES OF STRIPE (YELLOW) RUST IN BREAD WHEAT LANDRACES

**Alignment of seedling experiment QTLs to the previously identified YR genes/QTLs**

SNP1110537 in association with seedling resistance to *Warrior* pathotype was found within the genomic region of Yr genes *Yr15*, *YrCH52*, *Yr64,* and *YrAlp* (Cheng et al., 2014; Lin & Chen 2007). All these previously reported genes confer major gene or all-stage resistance to stripe rust. *Warrior* pathotype is avirulent to *Yr15* and therefore, SNP1110537 could represents *Yr*15. However, *PstS2* is also avirulent to *Yr*15 but the region was not detected in the experiment with *PstS2* and therefore it is possible that this region confers resistance due to some other gene hence further genetic analysis is required to confirm the relationship.

Two different genomic regions were identified on chromosome 2A in association with seedling resistance. Two loci SNP100467667 and SNP1110618 (**Supplemental Table** **S4)** were in the same chromosomal regions of previously identified two QTL *QYr.ucw‐2A.3* and *QYr.sun‐2AS_Kukri* respectively on the short arm of chromosome 2A (Maccaferri et al., 2015; Bariana et al., 2010). Two *Yr* genes *Yrxy2* and *Yr32* are also located in the same region. The *PstS2* is avirulent on the *Yr32* gene whereas seedling gene *Yrxy2* is a recessive high-temperature sensitive gene, therefore, we may conclude that resistance offered by this genomic region is due to *Yr32* as seedling tests were not subjected to any high-temperature stress. This statement is further validated by the non-significant association of this genomic region during the experiment with *Warrior* pathotype as it is virulent to the *Yr32* gene. SNP100462582 was in association with seedling resistance against the *Warrior* pathotype on chromosome 2A. According to the consensus maps (Bulli et al., 2016; Maccaferri et al., 2015), SNP100462582 lies within the genomic region of QTL *QYr.inra_2AL.2_CampRemy* (Boukhatem et al., 2002).

SNP2272062 was identified within the genomic region of *Pst* gene *Yr54* (McIntosh et al., 2014) and two previously reported QTL, *QYr.tam_2D_Quaiu* and *QYr.jic-2D_Briagdier* (Basnet et al., 2014; Jagger et al., 2011). *Pst* gene *Yr54* and *QYr.tam_2D_Quaiu* both confer APR whereas *QYr.jic-2D_Briagdier* confers seedling stripe rust resistance therefore, it is likely that resistance conferred by SNP2272062 is linked to *QYr.jic-2D_Briagdier.*

One QTL SNP 1102819 identified on chromosome 3A maps in the region where Bulli et al., (2016) and Hou et al., (2015) identified a seedling QTL. This region was found non-significantly associated in seedling experiments with *PstS2* pathotype as well as field experiments therefore we may conclude that this region contains race-specific seedling *Yr* gene(s) and/or QTL.

SNP1714660 maps in the same proximal region where Jighly et al., (2015) reported a QTL, *wPt-800213-IWA6510*, in Genome-Wide Association Mapping (GWAM) panel. However, *wPt-800213-IWA6510* was reported as an APR QTL while in our study SNP1714660 is a seedling resistant QTL thus, either SNP1714660 tags a different genomic region or most likely this region confers all stage race-specific resistance to stripe rust.

Three QTL, two for *Warrior* and one for *PstS2*, were identified on chromosome 3D for both pathotypes used in the study. SNP1228502 seems to be previously reported QTL *IWA3012_Seedling* (Zegeye et al., 2014). SNP3957546 and SNP1140683 identified on chromosome 3D are in association with seedling resistance to *Warrior* and *PstS2* pathotype. Based on the consensus map (Bulli et al., 2016 and Maccafferi et al., 2015) both SNPs seems to be in the region of *Yr45* however, we do not know the response of *Yr45* to the *PstS2* and *Warrior* race since the source of *Yr45* has not been tested against the two pathotypes and therefore further analysis may are required for clarifications. However, it is important to notice that this region confers resistance to both races used in the study hence, revealing the importance of the region for seedling resistance.

Two SNPs were detected on chromosome 4B for both *PstS2* and *Warrior* pathotype however at different genomic regions, therefore, likely tagging different QTLs. Stripe rust seedling resistance loci SNP1013303 was identified near the previously reported *Yr50* and *Yr62* genes and the QTL *QYr.sun‐4B_Janz* (Liu et al., 2013; Lu et al., 2014; Zwart et al., 2010). Since no source of either *Yr50* or *Yr62* was used in the differential sets therefore, further genetic analysis is necessary to conclude any relationship with these two genes.

SNP2262271 was significant in the region of already reported QTL *QYr.caas-6AL (IWB39473) Zhong 892* (Liu et al., 2015). QTL *QYr.caas-6AL (IWB39473)_Zhong 892* tags minor effect APR thus, it is likely that SNP2262271 and *QYr.caas-6AL (IWB39473)_Zhong 892* are not linked therefore further genetic analysis is required to confirm their relationship.

Two SNPs were identified to be associated with both the pathotypes used in the study however, tagging different genomic regions hence, linked to different QTL and/or *Yr* gene(s). SNP4990334 was found in association with *Yr35* and *QYr.ufs‐6B_Kariega* (Prins et al., 2011; McIntosh et al., 2014). We lack the information whether both the pathotypes used in the study are either virulent or avirulent on the *Pst* gene *Yr35* thus, further investigations are necessary to confirm whether the resistance conferred by SNP4990334 is due to *Yr35*. Prins et al., (2011) reported minor seedling and adult plant effects of *QYr.ufs‐6B_Kariega* therefore, it is possible that SNP4990334 is linked to the QTL as well but further investigations will confirm the relationship. Seedling resistance loci SNP991646 was found overlapping with *QYr.ucw‐6B (IWA7257)* (Maccaferri et al., 2015). However, the QTL *QYr.ucw‐6B (IWA7257)* was not resistant to any of the races used by Maccaferri et al., (2015) while the virulence formulas for all the pathotypes used in that study were similar to *PstS2* therefore, and it is likely that SNP991646 tags a different genomic region. Further genetic analysis is required to identify their relationship. A *Pst* gene *Yr36* and four other previously reported QTL (Bulli et al., 2016; Maccaferri et al., 2015; Bariana et al., 2010; Uauy et al., 2005; Suenaga et al., 2003) were in the same region as SNP100451841 (**Supplemental Table** **S4)**. It is important to note that an APR SNP1242315 was identified in the same genomic region as of SNP991646, highlighting the importance of this region as a potential seedling and APR resistance QTL.

**Alignment of field experiment QTLs to the previously identified YR genes/QTLs**

SNP1252697 lies on 498,822,949 bp position on chromosome 1A which seems similar to *QYr.sun‐1A_Janz* reported by Bariana et al., (2010). The *QYr.sun‐1A_Janz* is an adult plant resistance QTL therefore it is likely that SNP1252697 resistance is linked to *QYr.sun‐1A_Janz*.

SNP100495600 detected in this study is in the same genomic regions of previously identified *Yr29* gene and a QTL (William et al., 2003; Melichar et al., 2008). Based on the field response, *Yr29* and SNP100495600 seem to be related.

SNP3935538 detected in this study is found to be in the same region as previously identified QTL *wPt-6271* and *QYr.inra-2BS_Renan* (Jighly et al., 2015; Dedryver et al., 2009). However, Jighly et al., (2015) reported *wPt-6271* as a seedling resistance QTL therefore, it is possible that *wPt-6271* and SNP3935538 are tagging different genomic regions and QTL.

Three APR associated SNPs on chromosome 2D were identified in this study, SNP1125359 is within proximal distances to *Yr55* and *QYr.tam_2D_Quaiu* (McIntosh et al., 2014; Basnet et al., 2014). SNP1071080 lies near the genomic region of *Yr54* (McIntosh et al., 2014). Both *Yr54* and *Yr55* are reported as APR genes, therefore, it is likely that APR resistance conferred due to the SNPs identified in this study is related to these genes however, further genetic analyses are required to establish the relationship between the SNPs and the genes. Two previously reported QTLs on chromosome 2D, *QYr.caas-2DS_Libellulaz* and *QYr.wpg-2D.1 (IWA1939)* (Lu et al., 2009; Naruoka et al., 2015), exhibit APR effect and are in the same region as SNP100353195 therefore, it is likely that this region confers APR response.

Several QTLs are reported on chromosome 3B of which *QYr.cim‐3B_Pastor* and *QRYr3B.2* (Jighly et al., 2015; Rosewarne et al., 2012) are in the region of SNP1863248 identified in this study. The two QTLs reported earlier conferred APR effect therefore it is likely that SNP1863248 is linked to these QTLs.

Basnet et al., (2014) reported a minor effect APR QTL, *QYr.tam‐3D_Quaiu* on chromosome 3D which seems to be in the region of SNP986647 however, SNP986647 is a major effect APR QTL therefore, it is possible either these two QTLs are linked or tag different genomic regions because on subset data the *QYr.tam‐3D_Quaiu* became moderate effect APR QTL, thus further genetic analysis is required to confirm their relationship.

SNP2282076 associated with APR on chromosome 4A lies in the same genomic region as previously identified APR QTL *IWA3756_APR* (Zegeye et al., 2014).

SNP1200934 was found associated with APR to *Pst* on chromosome 4B. Two *Pst* genes, *Yr50* and *Yr62* (Liu et al., 2013 and Lu et al., 2014) and an APR QTL, *QYr.wpg-4B.2 (IWA3994)* (Naruoka et al., 2015) are mapped on this region. Naruoka et al., (2015) reported *QYr.wpg-4B.2 (IWA3994)* as an APR QTL therefore it is possible that SNP identified in this study might be in linkage with *QYr.wpg-4B.2 (IWA3994)*.

SNP1051014 lies within the region of *QYr-5A_Opata85* and *QYr.cim-5AL_Pastor* on the long arm of chromosome 5A (Rosewarne et al., 2012; Boukhatem et al., 2002). Another locus detected on the same chromosome SNP1019889 lies within the genomic region previously identified gene *Yr48* and an APR QTL *QYrdr.wgp-5AL (IWA2646)* (Lowe et al., 2011; Hou et al., 2015)*.* SNP1184257 locus is in the same region as *Yr48,* *QYr.ucw-5A.1* and *QYr.ucw-5AL_PI610750* (Lowe et al., 2011; Maccaferri et al., 2015). However, its relationship with *Yr48* needs further genetic analysis to be confirmed.

SNP4991320 on chromosome 5B lies within *QYr-5B_Oligoculm* (Suenaga et al., 2003). Both *QYr-5B_Oligoculm* and SNP4991320 show minor effect APR resistance, therefore, they are likely to be linked with each other.

APR SNP1242315 was found in close proximity to *QYr.ucw‐6B (IWA7257)* and *QYr.wsu-6B.1 (IWA7257)* (Bulli et al., 2016; Maccaferri et al., 2015). The QTLs *QYr.ucw‐6B (IWA7257)* and *QYr.wsu-6B.1 (IWA7257)* were reported seedling susceptible adult plant resistant QTLs although, SNP1242315 is identified in this study as only APR QTL however, another major effect seedling QTL SNP991464 in association with seedling resistance to *PstS2* is located at approximately 2.5 cM distance from SNP1242315 both SNPs are not in LD with each other, therefore, tagging different genomic regions. Furthermore, BLAST analysis revealed a putative *R*-gene *TraesCS6B01G384000* as well as a water stress-related gene TraesCS6B01G383800 encoding Dehydrin-LEA (late embryogenesis abundant) protein was also identified in the same genomic region indicating an important genomic region for breeding for biotic and abiotic stresses.

APR QTL SNP 2264230 showed association with field resistance and was tagged in the genomic region of *QYr.sgi-7A_Kariega* (Prins et al., 2011). *QYr.sgi-7A_Kariega* is reported as a minor APR QTL and it seems SNP 2264230 and *QYr.sgi-7A_Kariega* are linked.

**References:**

Bariana, H. S., Bansal, U. K., Schmidt, A., Lehmensiek, A., Kaur, J., Miah, H., ... & McIntyre, C. L. (2010). Molecular mapping of adult plant stripe rust resistance in wheat and identification of pyramided QTL genotypes. *Euphytica*, *176*(2), 251-260.

Bariana, H. S., Bansal, U. K., Schmidt, A., Lehmensiek, A., Kaur, J., Miah, H., ... & McIntyre, C. L. (2010). Molecular mapping of adult plant stripe rust resistance in wheat and identification of pyramided QTL genotypes. *Euphytica*, *176*(2), 251-260.

Basnet, B. R., Singh, R. P., Ibrahim, A. M. H., Herrera-Foessel, S. A., Huerta-Espino, J., Lan, C., & Rudd, J. C. (2014). Characterization of Yr54 and other genes associated with adult plant resistance to yellow rust and leaf rust in common wheat Quaiu 3. *Molecular breeding*, *33*(2), 385-399.

Boukhatem, N., Baret, P. V., Mingeot, D., & Jacquemin, J. M. (2002). Quantitative trait loci for resistance against yellow rust in two wheat-derived recombinant inbred line populations. *Theoretical and Applied Genetics*, *104*(1), 111-118.

Bulli, P., Zhang, J., Chao, S., Chen, X., & Pumphrey, M. (2016). Genetic architecture of resistance to stripe rust in a global winter wheat germplasm collection. *G3: Genes, Genomes, Genetics*, *6*(8), 2237-2253.

Cheng, P., Xu, L. S., Wang, M. N., See, D. R., & Chen, X. M. (2014). Molecular mapping of genes Yr64 and Yr65 for stripe rust resistance in hexaploid derivatives of durum wheat accessions PI 331260 and PI 480016. *Theoretical and applied genetics*, *127*(10), 2267-2277.

Dedryver, F., Paillard, S., Mallard, S., Robert, O., Trottet, M., Nègre, S., Verplancke, G. and Jahier, J., 2009. Characterization of genetic components involved in durable resistance to stripe rust in the bread wheat ‘Renan’. *Phytopathology*, *99*(8), pp.968-973.

Hou, L., Chen, X., Wang, M., See, D. R., Chao, S., Bulli, P., & Jing, J. (2015). Mapping a large number of QTL for durable resistance to stripe rust in winter wheat Druchamp using SSR and SNP markers. *PLoS One*, *10*(5), e0126794.

Jagger, L. J., Newell, C., Berry, S. T., MacCormack, R., & Boyd, L. A. (2011). The genetic characterisation of stripe rust resistance in the German wheat cultivar Alcedo. *Theoretical and applied genetics*, *122*(4), 723-733.

Jighly, A., Oyiga, B. C., Makdis, F., Nazari, K., Youssef, O., Tadesse, W., ... & Ogbonnaya, F. C. (2015). Genome-wide DArT and SNP scan for QTL associated with resistance to stripe rust (Puccinia striiformis f. sp. tritici) in elite ICARDA wheat (Triticumaestivum L.) germplasm. *Theoretical and applied genetics*, *128*(7), 1277-1295.

Lin, F., & Chen, X. M. (2007). Genetics and molecular mapping of genes for race-specific all-stage resistance and non-race-specific high-temperature adult-plant resistance to stripe rust in spring wheat cultivar Alpowa. *Theoretical and Applied Genetics*, *114*(7), 1277-1287.

Liu, J., Chang, Z., Zhang, X., Yang, Z., Li, X., Jia, J., ... & Wang, J. (2013). Putative Thinopyrum intermedium-derived stripe rust resistance gene Yr50 maps on wheat chromosome arm 4BL. *Theoretical and Applied Genetics*, *126*(1), 265-274.

Liu, J., He, Z., Wu, L., Bai, B., Wen, W., Xie, C., & Xia, X. (2015). Genome-wide linkage mapping of QTL for adult-plant resistance to stripe rust in a Chinese wheat population Linmai 2× Zhong 892. *PloS one*, *10*(12), e0145462.

Lowe, I., Cantu, D., & Dubcovsky, J. (2011). Durable resistance to the wheat rusts: integrating systems biology and traditional phenotype-based research methods to guide the deployment of resistance genes. *Euphytica*, *179*(1), 69-79.

Lu, Y., Lan, C., Liang, S., Zhou, X., Liu, D., Zhou, G., ... & He, Z. (2009). QTL mapping for adult-plant resistance to stripe rust in Italian common wheat cultivars Libellula and Strampelli. *Theoretical and Applied Genetics*, *119*(8), 1349-1359.

Lu, Y., Wang, M., Chen, X., See, D., Chao, S., & Jing, J. (2014). Mapping of Yr62 and a small-effect QTL for high-temperature adult-plant resistance to stripe rust in spring wheat PI 192252. *Theoretical and Applied Genetics*, *127*(6), 1449-1459.

Maccaferri, M., Zhang, J., Bulli, P., Abate, Z., Chao, S., Cantu, D., ... & Dubcovsky, J. (2015). A genome-wide association study of resistance to stripe rust (Puccinia striiformis f. sp. tritici) in a worldwide collection of hexaploid spring wheat (Triticum aestivum L.). *G3: Genes, Genomes, Genetics*, *5*(3), 449-465.

McIntosh, R. A., Dubcovsky, J., Rogers, J. W., Morris, C. F., Appels, R., & Xia, X. (2014). Catalogue of gene symbols for wheat: 2013-14 Supplement. *Annual Wheat Newsletter*, *58*.

Melichar, J. P. E., Berry, S., Newell, C., MacCormack, R., & Boyd, L. A. (2008). QTL identification and microphenotype characterisation of the developmentally regulated yellow rust resistance in the UK wheat cultivar Guardian. *Theoretical and Applied Genetics*, *117*(3), 391.

Naruoka, Y., Garland-Campbell, K. A., & Carter, A. H. (2015). Genome-wide association mapping for stripe rust (Puccinia striiformis F. sp. tritici) in US Pacific Northwest winter wheat (Triticum aestivum L.). *Theoretical and Applied Genetics*, *128*(6), 1083-1101.

Prins, R., Pretorius, Z. A., Bender, C. M., & Lehmensiek, A. (2011). QTL mapping of stripe, leaf and stem rust resistance genes in a Kariega× Avocet S doubled haploid wheat population. *Molecular breeding*, *27*(2), 259-270.

Rosewarne, G. M., Singh, R. P., Huerta-Espino, J., Herrera-Foessel, S. A., Forrest, K. L., Hayden, M. J., & Rebetzke, G. J. (2012). Analysis of leaf and stripe rust severities reveals pathotype changes and multiple minor QTLs associated with resistance in an Avocet× Pastor wheat population. *Theoretical and Applied Genetics*, *124*(7), 1283-1294.

Suenaga, K., Singh, R. P., Huerta-Espino, J., & William, H. M. (2003). Microsatellite markers for genes Lr34/Yr18 and other quantitative trait loci for leaf rust and stripe rust resistance in bread wheat. *Phytopathology*, *93*(7), 881-890.

Uauy, C., Brevis, J. C., Chen, X., Khan, I., Jackson, L., Chicaiza, O., ... & Dubcovsky, J. (2005). High-temperature adult-plant (HTAP) stripe rust resistance gene Yr36 from Triticum turgidum ssp. dicoccoides is closely linked to the grain protein content locus Gpc-B1. *Theoretical and Applied Genetics*, *112*(1), 97.

William, M., Singh, R. P., Huerta-Espino, J., Islas, S. O., & Hoisington, D. (2003). Molecular marker mapping of leaf rust resistance gene Lr46 and its association with stripe rust resistance gene Yr29 in wheat. *Phytopathology*, *93*(2), 153-159.

Zegeye, H., Rasheed, A., Makdis, F., Badebo, A., & Ogbonnaya, F. C. (2014). Genome-wide association mapping for seedling and adult plant resistance to stripe rust in synthetic hexaploid wheat. *PLoS One*, *9*(8), e105593.

Zwart, R. S., Thompson, J. P., Milgate, A. W., Bansal, U. K., Williamson, P. M., Raman, H., & Bariana, H. S. (2010). QTL mapping of multiple foliar disease and root-lesion nematode resistances in wheat. *Molecular Breeding*, *26*(1), 107-124.
