# Supplementary material for: Genome‐wide association study of resistance to PstS2 and Warrior races of Puccinia striiformis f. sp. tritici (stripe rust) in bread wheat landraces
Source: Plant Genome. 2020 Dec 7;14(1):e20066. doi: 10.1002/tpg2.20066 (PMC12807014; doi:10.1002/tpg2.20066)
Supplement: Supplementary file 2 [file TPG2-14-e20066-s009.docx]

S2. Table: Virulence/Avirulence formula for the *PstS2* and *Warrior* Pathotypes of Pst.

| **Pathotype** | **Avirulence formula** | **Virulence formula** |
| --- | --- | --- |
| Warrior | *Yr5, Yr10, Yr15, Yr24, Yr27* | *YrA, YrAvS, Yr1, Yr2, Yr3, Yr4, Yr6, Yr7, Yr8, Yr9, Yr17, Yr25,Yr32, YrSp, YrSu, YrND, YrSD,YrTres* |
| PstS2 | *Yr1, Yr3, Yr4, Yr5, Yr10, Yr15, Yr17, Yr24, Yr32, YrSp, YrND, YrSD, YrTres* | *YrA, YrAvS, Yr2, Yr6, Yr7, Yr8, Yr9, Yr25, Yr27, YrSu* |
